# *Streptomyces shaoguanensis* sp. nov.: elucidating the mechanisms of efficient chicken feather degradation and its potential for biofertilizer development

**DOI:** 10.1186/s12934-025-02878-8

**Published:** 2025-11-28

**Authors:** Di Zhou, Weibin Zheng, Yijie Li, Ziqi Zhang, Xia Ding, Ye Ke

**Affiliations:** 1 School of Life Sciences, Nanchang University, Nanchang, 330031 China; 2 School of Biology and Agriculture, Shaoguan University, Shaoguan, 512005 China; 3 Jiangxi Province Key Laboratory of Drug Target Discovery and Validation, Nanchang University, Nanchang, 330031 China

**Keywords:** *Streptomyces shaoguanensis* sp. nov., High-efficiency feather degradation, Degradation mechanism, Plant growth promotion

## Abstract

**Background:**

Feather waste, a byproduct of the poultry industry, remains underutilized due to its recalcitrant nature. While microbial conversion holds substantial potential, the scarcity of high-efficiency degrading strains hampers industrial application.

**Results:**

A novel feather-degrading actinobacterium, designated KK^T^, exhibited highly efficient decomposition of feather waste. When cultured with 10% (w/v) chicken feathers as sole nutrient source, it achieved over 50% degradation within 8 days. Taxonomic characterization identified strain KK^T^ as a novel species of the genus *Streptomyces*, with the proposed name *Streptomyces shaoguanensis* sp. nov.. Genomic analysis of strain KK^T^ revealed an abundance of functionally uncharacterized genetic elements and 26 predicted biosynthetic gene clusters (BGCs) for secondary metabolites. Integrated transcriptomic and biochemical analyses suggested that feather degradation by *S. shaoguanensis* KK^T^ represents an adaptive physiological response. This process was found to sustain an alkaline fermentation environment through continuous ammonia release and to efficiently disrupt disulfide bonds via a non-sulfite-dependent mechanism mediated by cysteine, H₂S and reductases. Simultaneously, highly efficient degradation was achieved through the temporally coordinated action of multiple proteases. Furthermore, when applied as a biofertilizer, the feather hydrolysate significantly promoted the growth of *Brassica rapa* subsp. *chinensis* (Pak Choi) compared to commercial amino acid fertilizers, achieving 13.1% higher fresh weight, 14.4% greater leaf area, 16.3% increased chlorophyll content, and 45.3% elevated soluble protein levels.

**Conclusions:**

Here, a novel *Streptomyces* species strain KK^T^ with superior feather-degrading efficiency was reported. A wealth of functionally uncharacterized genes and significant biosynthetic potential in the genome of strain KK^T^ laid a genetic groundwork for the exploration of its novel physiological functions and the discovery of uncharacterized metabolites. Integrative analyses of genomics, transcriptomics, and biochemical profiles of the degradation metabolites, together, uncovered the underlying mechanism of superior feather-degrading capacity. Additionally, the feather hydrolysate demonstrated a significant growth-promoting effect on Pak Choi. This finding provides a solid foundation for the sustainable valorization of feather waste and the development of novel biofertilizers.

**Supplementary Information:**

The online version contains supplementary material available at 10.1186/s12934-025-02878-8.

## Background

Feather waste is rich in protein, minerals, vitamins, and growth factors, making it highly promising for conversion into feed, fertilizers, and industrial raw materials [1–4]. USDA data show global broiler production reached 103.0 million metric tons (MMT) in 2024, with a projected rise to 104.9 MMT in 2025. At this scale, annual feather waste generation is estimated at 5.2–7.5 MMT. Feathers owe their exceptional mechanical stability to a keratin-dominated composition. This stability originates from keratin’s tightly packed β-sheet structure, which is extensively cross-linked via disulfide bonds, hydrogen bonds, and hydrophobic interactions, thus rendering it highly resistant to degradation by conventional proteases (e.g., pepsin, papain) and hence poorly utilized [5–8]. Most feather waste is currently landfilled or incinerated, with only small fractions recycled via physical (e.g., steam pressure cooking), chemical (e.g., strong-acid/alkali treatment), or mechanical processes (e.g., grinding). These methods are hindered by inherent limitations: high operational costs, low efficiency, and the generation of poorly soluble protein hydrolysates of low nutritional value (due particularly to the degradation of essential amino acids), all of which result in substantial resource waste and environmental pollution [6, 9, 10]. It is therefore imperative to develop eco-friendly bioprocessing technologies.

Herein, microbial degradation has emerged as a compelling solution. This process leverages feather-degrading microorganisms that produce a suite of enzymes including keratinases, lipolytic enzymes, and disulfide bond reductases. Through synergistic action, these enzymes efficiently break down recalcitrant feather keratin into value-added products such as soluble proteins, peptides, and amino acids [11], offering an economical and eco-friendly conversion pathway. Although diverse feather-degrading bacteria [12–19] and fungi [8] have been isolated, most show high degradation efficiency only with low-concentration feather substrates (≤ 1.0%, w/v). A sharp decline in efficiency is observed at high concentrations [20]. For instance, *Streptomyces netropsis* A-ICA achieved 84.0 ± 2.0% degradation using 1.0% (w/v) feathers after 7 days under optimized conditions [19], while *Streptomyces exfoliatus* CFS 1068 exhibited a notable decline from 96.6% degradation at 0.5% feather substrate to merely 72.1% at 2.0% [21]. This poor performance with concentrated substrates poses a major barrier to industrial-scale implementation. Consequently, discovering novel strains capable of efficient degradation under high-feather-load conditions is an urgent priority.

Several theories have been proposed to explain microbial feather degradation mechanisms, including enzymatic hydrolysis, thiolysis, mechanical pressure, and redox theories [22, 23]. Current research prioritizes enzymatic hydrolysis and thiolysis mechanisms, notably sulfite-mediated thiolysis. For instance, using multi-omics techniques, Li et al. identified disulfide bond reduction as the initial critical step in feather degradation by *Streptomyces* sp. SCUT-3, with upregulated *cdo* (cysteine dioxygenase) and *tauE* (sulfite exporter) genes suggesting sulfite involvement in disulfide bond cleavage [18]. Peng et al. further emphasized sulfite’s role in initiating keratinase catalysis [24]. Through metagenomic annotation, Kang et al. predicted sulfide/GSH-dependent disulfide reduction mechanisms [23]. Under sulfur starvation, Jin et al. observed that *Fervidobacterium islandicum* AW-1 achieved efficient keratin degradation via a high-fidelity SufS-to-SufU transfer system, proposing that coupling intracellular redox homeostasis with extracellular cysteine-cystine cycling was essential for degradation [25]. Despite these advances, inter-strain mechanistic variations persist. For example, when degrading wool keratin, *S. fradiae* showed no detectable sulfite, cysteine desulfurization, or H₂S excretion, providing no evidence for sulfite-mediated decomposition [26]. These findings imply undiscovered keratinolytic pathways. Thus, elucidating strain-specific degradation strategies will advance understanding of metabolic diversity in feather keratin decomposition and enable targeted strain development.

A novel strain KK^T^, isolated from chicken feather waste and identified as a new *Streptomyces* species via polyphasic taxonomy, exhibits highly efficient degradation of elevated-concentration feathers (4.0–10.0% w/v). Temporal changes in the biochemical profile of the fermentation broth were monitored throughout the feather degradation process, including the dynamics of amino acids, soluble proteins, free thiols, protease activity, and sulfur metabolites. Integrated genomic-transcriptomic analyses demonstrated that this process represents an adaptive physiological response: a metabolic reprogramming triggered to counteract extracellular stress from high-concentration keratin. Critically, the feather hydrolysate served as an effective amino acid fertilizer, promoting the growth of *Brassica rapa* subsp. *chinensis* (Pak Choi). As a novel keratinolytic actinomycete, *Streptomyces* sp. KK^T^ not only expands the phylogenetic diversity of feather-degrading streptomycetes, but also provides a promising strain and mechanistic insights for poultry waste valorization. Its application in amino acid fertilizer production further establishes a sustainable circular economy model for feather waste.

## Methods

### Material and strains

Chicken feather waste was obtained from a poultry trading market in Shaoguan City, Guangdong Province (23.08°–25.52°N, 112.88°–114.75°E). The feathers were rinsed with tap water, air-dried, and reserved for later use.

The strains *Streptomyces zaomyceticus* CGMCC 4.1953^T^ and *S. exfoliatus* GDMCC 4.124^T^ were obtained from China General Microbiological Culture Collection Center (CGMCC) and Guangdong Microbial Culture Collection Center (GDMCC), respectively.

A commercially sourced amino acid-containing water-soluble fertilizer (Guangdong Jiangmen Plant Protection Co., Ltd., China), with the following specifications: amino acid content ≥ 100 g/L; macronutrients (N-P-K) ≥ 20% (w/v), comprising 80% readily available nitrogen and 20% organic nitrogen; trace elements (Cu, Mn, Zn, and B) ≥ 20 g/L; sulfur: an appropriate amount; and pH 6.2 ± 1.0. Prior to application, the fertilizer was diluted 500–1000 fold with deionized water.

### Isolation of keratinolytic microbial strains

Freshly collected chicken feather waste (10 g) was inoculated into 100 mL of enrichment medium in 500 mL flasks and incubated at 30 °C with shaking at 150 rpm for 3 days. The enrichment medium contained per liter: NaCl 0.5 g, NH_4_Cl 0.5 g, KH_2_PO_4_ 0.4 g, K_2_HPO_4_ 0.3 g, MgCl_2_ 0.1 g, yeast extract 1.0 g, and chicken feather powder 10.0 g, with pH adjusted to 7.2–7.5 before sterilization. Subsequently, an aliquot of the enriched culture was subjected to 10-fold serial dilution and spread-plated onto feather meal agar medium. The medium contained (per liter) K_2_HPO_4_ 1.5 g, FeSO_4_ 0.015 g, MgSO_4_·7H_2_O 0.025 g, CaCl_2_ 0.025 g, chicken feather powder 10.0 g and agar 15.0 g. It was prepared in ddH_2_O, brought to a final volume of 1 L, and adjusted to pH 7.2–7.5. After incubation at 30 °C for 7 days, single colonies were picked based on rapid growth, high colony density, and well-developed aerial hyphae, and then streaked onto fresh feather meal agar plates. Streaking was repeated until pure cultures were obtained.

Purified isolates were cultured in Tryptic Soy Broth (TSB) at 30 °C with shaking (150 rpm) for 48 h, and cells were harvested by centrifugation. The chicken feather fermentation medium was prepared by adding 0.4 g of chicken feathers to a 500 mL flask, followed by 100 mL of a basal salts solution (containing per liter: K_2_HPO_4_ 1.5 g, FeSO_4_ 0.015 g, MgSO_4_·7H_2_O 0.025 g, CaCl_2_ 0.025 g; pH adjusted to 7.2–7.5). The medium was then sterilized at 121 °C for 20 min. Cells were inoculated into the sterile medium at 1 × 10⁷ CFU/mL and incubated under the same conditions (30 °C, 150 rpm) for 8 days. Undegraded feather residues were filtered out through Whatman No. 1 filter paper, dried at 60 °C to constant weight, and weighed. The percentage of degradation was calculated as: [(Initial dry weight – Residual dry weight)/Initial dry weight] × 100. All experiments were performed in triplicate. The strain exhibiting the highest degradation percentage was selected for subsequent studies.

### Phylogenetic tree construction based on 16S rRNA gene sequence

Genomic DNA of strain KK^T^ was extracted using a modified rapid DNA isolation protocol originally described for yeast [27]. Briefly, mycelia from a 48-hour culture grown in ISP2 liquid medium (2–4 mL) were harvested by centrifugation (at 12,000 ×g, 1 min, room temperature). The pellet was resuspended in 50 µL of STES buffer (0.2 M Tris-Cl [pH 7.6], 0.5 M NaCl, 0.01 M EDTA, 0.01% SDS). After the addition of 50 µL of acid-washed glass beads (0.4 mm; Sigma) and 20 µL of TE buffer (10 mM Tris-Cl, 1 mM EDTA; pH 7.6), the cells were lysed by vigorous vortexing (5 min) with 60 µL of phenol: chloroform (1:1, v/v; freshly prepared). Following centrifugation (12,000 ×g, 5 min), the aqueous phase was transferred to a new 1.5 mL microcentrifuge tube. Nucleic acids were precipitated with 2.5 volumes of absolute ethanol and 0.1 volume of 3.0 M sodium acetate (pH 5.2) on ice for 15 min. The DNA pellet, collected by centrifugation (12,000 ×g, 10 min, 4 °C), was washed with 100 µL of 70% ethanol, recentrifuged for 5 min, and the supernatant was discarded. The pellet was air-dried at room temperature and finally resuspended in 40 µL of TE buffer. The 16S rRNA gene was PCR-amplified from this genomic DNA template using primers 8–27 F and 1523-1504R and sequenced [28]. The resulting sequence was subjected to comparative analysis on the EzBioCloud platform (https://www.ezbiocloud.net), where the twenty-four top-hit sequences from type strains of phylogenetically related species were retrieved. Multiple sequence alignment was performed using CLUSTAL X2.0 [29], followed by phylogenetic tree construction in MEGA 6.0 software [30] employing both the neighbor-joining (NJ) [31] and maximum likelihood (ML) algorithms [32]. Bootstrap support was assessed with 1000 replications, with other parameters set to default [33]. *Kitasatospora cystarginea* JCM 7356^T^ was designated as the outgroup reference strain.

### Phenotypic characterization of strain KK^T^

Strain KK^T^ was individually streaked onto ISP1–7 (International Streptomyces Project formulations, ISP) media, nutrient agar (NA), Czapek’s sucrose agar (CZ), and potato dextrose agar (PDA), and incubated at 30 °C. Cultural characteristics, including aerial and substrate mycelia coloration, as well as soluble pigment production were documented after7, 14, and 21 days. Concurrently, 14-day-old surface cultures on ISP2 agar (30 °C) were used for morphological characterization via scanning electron microscopy (SEM; Thermo Scientific Apreo 2 S, USA).

Strain KK^T^ was streaked onto ISP2 agar plates and incubated at 5–43 °C for 14 days to determine its growth temperature range and optimal temperature. For determining growth pH range and optimal pH, the strain was individually inoculated into ISP2 liquid media adjusted to pH 3.0–12.0 [34], followed by incubation at 30 °C with shaking at 150 rpm. Salt tolerance was evaluated in ISP2 broth supplemented with gradient NaCl concentrations (0–6.0% w/v) under the same incubation conditions. Carbon and nitrogen source utilization assays followed the protocols described by Shirling & Gottlieb (1966) [35] and Williams et al. (1983) [36]. Physiological and biochemical characterization included assays for sugar fermentation; catalase and oxidase activity; coagulation and peptonization of milk; nitrate reduction; hydrolysis of esculin, gelatin, starch, and Tween 80; urease production; and utilization of organic acid salts. Extracellular enzyme profiles were analyzed using API ZYM kits (bioMérieux, France) [37].

### Chemotaxonomic studies

Strain KK^T^ was cultured in ISP2 liquid medium (30 °C, 150 rpm, 5 days). Mycelia were harvested by centrifugation, washed three times with sterile saline (0.85% NaCl), and lyophilized for chemotaxonomic analyses. Whole-cell sugar profiles and cell wall peptidoglycan diamino acids were analyzed by thin-layer chromatography (TLC) according to Lechevalier & Lechevalier (1980) [38] and Hasegawa et al. (1983) [39], respectively. Polar lipids were extracted following Minnikin et al. (1984) [40] and separated by two-dimensional TLC (silica gel, 10 × 10 cm; Merck). Fatty acids were analyzed using an Agilent 6890 gas chromatograph (FID detector) on a 1707 column with the MIDI Sherlock system [41]. Quinones were extracted as described by Minnikin et al. (1984) [40] and analyzed by high-performance liquid chromatography (HPLC; Agilent 1200) on a Zorbax Eclipse XDB-C18 column (5 μm, 250 × 4.6 mm) with a methanol-isopropanol (2:1 v/v) mobile phase (1.0 mL/min, detected at 270 nm).

### Genome sequencing, assembly, annotation and phylogenomic analysis of strain KK^T^

Genomic DNA of strain KK^T^ underwent paired-end sequencing on the Illumina HiSeq 2500 and PacBio RS II platforms (Sangon Biotech, Shanghai). Raw data were filtered and hybrid-assembled using Unicycler v0.5.1 [42], followed by gap filling (GapFiller v2.1.2) [43], error correction (Pilon v1.24) [44], and completeness assessment (BUSCO v5.8.3) [45] to generate a high-quality genome assembly. Gene prediction via Prokka v1.14.6 [46] identified coding sequences (CDS), tRNAs, and rRNAs. Functional annotation was carried out using the NR, RefSeq, SwissProt, and Clusters of Orthologous Groups (COG) databases [47], while Gene Ontology (GO) terms were assigned using InterProScan v5.75–106.0.0 [48]. Kyoto Encyclopedia of Genes and Genomes (KEGG) mapping was performed using KAAS (https://www.genome.jp/) with the BBH method [49]. Secondary metabolite biosynthetic gene clusters (BGCs) were predicted using antiSMASH v7.1.0 [50]. Signal peptides were predicted by SignalP 6.0 [51]. Protein sequences were aligned against the MEROPS database [52] via Diamond v2.1.9 [53] (E-value ≤ 1 × 10⁻⁵) for classification. Putative keratinases (S08/M04 peptidases) were compared with reference serine-type keratinases (KerA, accession: AAB34259; KerC, ABY65903; KerF, ADK74828; KerQ7, AKN20219) [23] and the metal-type keratinase AJD77429 [54] to identify potential candidates.

Digital DNA-DNA hybridization(dDDH) values were calculated using Genome-to-Genome Distance Calculator (GGDC) v3.0 (Formula 2) [55]. Average nucleotide identity (ANI) was computed with the ANI Calculator (https://www.ezbiocloud.net/tools/ani) [56]. Whole-genome phylogeny was reconstructed using the Type Strain Genome Server (TYGS) [57].

### Transcriptome sequencing and biochemical characterization of feather degradation by strain KK^T^

Strain KK^T^ was inoculated into a chicken feather fermentation medium without additional sulfur source and incubated at 30 °C with shaking (150 rpm). The medium was prepared as previously described, with the modification that the basal salts solution contained FeCl_2_·4H_2_O (0.011 g/L) and MgCl_2_·6H_2_O (0.021 g/L) in place of FeSO₄ and MgSO₄·7H₂O. For transcriptomic analysis, cultures harvested at 0, 24, 48, and 96 h were centrifuged (12,000 ×g, 10 min, 4 °C). Microbial pellets were washed three times with sterile water, flash-frozen in liquid nitrogen, and submitted to Personal Biotechnology Co., Ltd (Shanghai, China) for Illumina PE150 sequencing. Raw sequencing data in FASTQ format were processed with fastp (v0.22.0) [58] for quality control, yielding high-quality clean reads. These reads were then aligned to the *de novo* assembled genome of strain KK^T^ from this study using Bowtie2 (v2.4.1) [59]. Gene expression levels were quantified with HTSeq (v0.9.1) [60]. For visualization and comparative purposes, expression levels were normalized using FPKM (Fragments Per Kilobase per Million fragments), while differential expression analysis between conditions was performed with DESeq2 (v1.38.3) [61]. Significantly differentially expressed genes were defined by a threshold of | log_2_ (Fold Change) | >1 and an adjusted p-value < 0.05. Concurrently, culture supernatants were collected at 24-hour intervals and clarified by centrifugation (12,000 ×g, 10 min, 4 °C). The resulting supernatants were subjected to comprehensive biochemical analyses. Protease activity was assayed by the Folin-Ciocalteu method [62], and amino acid content was determined via the ninhydrin assay [63]. Soluble protein concentration was measured using a modified bicinchoninic acid (BCA) kit (Sangon Biotech, Shanghai, China) with bovine serum albumin (BSA) standards. Thiol groups were quantified via the 5,5´-Dithiobis (2-nitrobenzoic acid) (DTNB) method [64], while free cysteine/cystine levels were analyzed through the acid-ninhydrin assay [65]. Disulfide bonds were measured employing the disodium 2-nitro-5-thiosulfobenzoate (NTSB) method [66], and free sulfite was quantified using a total and free sulfite assay kit (Megazyme, Ireland) with a free sulfite calibration curve. Sulfate quantification was performed using the barium chromate spectrophotometric method: 1 mL of supernatant was diluted to 25 mL with ultrapure water, acidified with 0.5 mL of 2.5 mol/L HCl, and boiled for 5 min. Then, 1.25 mL of acidified barium chromate solution (prepared by dissolving 2.5 g barium chromate in 2.5 mol/L HCl and bringing to a final volume of 100 mL) was added, followed by reboiling for 5 min. After cooling to room temperature, ammonia solution (25% [v/v] aqueous ammonia diluted 1:1 with ultrapure water) was added dropwise until the solution turned lemon-yellow, with two additional drops. The mixture was centrifuged (12,000 ×g, 10 min), and the absorbance of the clarified supernatant was measured at 420 nm. A calibration curve (0–0.2 mg/mL) using sodium sulfate solutions was constructed for quantification. The presence of gaseous products was detected qualitatively using lead acetate test paper (turns black upon exposure to H₂S) and red litmus paper (turns blue in the presence of NH₃), both suspended within the headspace of the fermentation flasks.

### SEM examination of chicken feather degradation

Native and degraded chicken feathers (degradation time points: 24, 48, 72, 120, 168, and 192 h) were collected separately, thoroughly rinsed with ultrapure water and air-dried at 50 ℃. Structural changes of feathers during degradation were examined using a scanning electron microscope (Hitachi TM3030, Hitachi High-Tech., Japan).

## Growth effects of chicken feather hydrolysate on *Brassica rapa* subsp. *chinensis* (Pak Choi)

### Production of chicken feather hydrolysate

Chicken feather hydrolysate (CFH) was produced by 10-day fermentation in 10% (w/v) chicken feather medium under conditions described previously, and the centrifuged supernatant was collected as the final CFH. Seventeen free amino acids in the CFH were quantified using method NY/T 1975–2010 (China’s Agricultural Industry Standard for water-soluble fertilizer analysis).

### Growth response of *Brassica rapa* subsp. *chinensis*(Pak Choi) in pot culture

The Pak Choi cultivation experiment was conducted in a naturally ventilated greenhouse at School of Biology and Agriculture, Shaoguan University, from late October to early December 2024, with no artificial control of light or humidity. The growth substrate, composed of peat moss (fertilizer-free, particle size 5–20 mm, pH 6.0), perlite, and vermiculite (both with a particle size of 3–6 mm), was blended at a 3:1:1 v/v ratio, moistened with 50% (v/w) water, and left overnight before being filled into 21.5 × 14.5 cm flowerpots (3 L each). Uniformly pre-germinated Pak Choi seeds were sown at a density of 15 seeds per pot. At the third true-leaf stage, seedlings were thinned to 5 uniform plants per pot. Three days post-thinning, foliar applications were initiated at 3-day intervals (50 mL per application). The treatments were as follows: (1) Control (CK): 0.3% (w/v) KH₂PO₄ solution; (2) CFH (T1): 0.3% (w/v) KH₂PO₄ solution supplemented with CFH (0.32 g/L total free amino acids); (3) Commercial fertilizer (T2): 0.3% (w/v) KH₂PO₄ solution supplemented with a commercial amino acid fertilizer (0.165 g/L total free amino acids). A total of 12 applications were made over 36 days prior to harvest, with five biological replicates per treatment (*n* = 5).

Pak Choi plants were harvested, rinsed with tap water, gently blotted dry with filter paper, and air-dried to remove surface moisture. Afterwards, the following per-plant parameters were recorded: (1) plant height (from soil surface to apical meristem); (2) shoot fresh weight (with roots excised at hypocotyl base); (3) total leaf number; and (4) leaf area of the third fully expanded leaf from the apex, quantified using ImageJ (v1.8.0, NIH, USA) under standardized lighting.

For soluble protein analysis, 0.5 g fresh mesophyll tissue (midribs excised) from the third fully expanded leaf was homogenized in 5 mL ddH_2_O. After centrifugation (10,000 ×g, 10 min, 4 ℃), soluble protein in the supernatant was quantified via Coomassie Brilliant Blue assay against BSA standards [67]. For chlorophyll extraction, the second fully expanded leaf from the apex was used: 0.5 g fresh mesophyll tissue (midribs excised) was homogenized with 0.10 g quartz sand, 0.05 g calcium carbonate powder, and 2 mL of ice-cold 80% (v/v) acetone. An additional 4 mL ice-cold 80% acetone was added, and grinding continued until the tissue was completely decolorized (pale white). After standing for 3–5 min, the mixture was centrifuged (10,000 ×g, 5 min, 4 ℃). The supernatant was transferred to a brown volumetric flask and adjusted to a final volume of 10 mL with ice-cold 80% acetone. Absorbance at 645 nm (A_645_) and 663 nm (A_663_) was measured to calculate total chlorophyll concentration (C, mg·L⁻¹) using Arnon’s equation:

$$C = {\text{ }}20.29A_{{645}} + {\text{ }}8.05A_{{663}}$$

Chlorophyll content (mg·g⁻¹ FW) was derived as:

$$ Chlorophyll\,content = \left( {C \times V} \right)/\left( {FW \times {\text{ }}1000} \right) \times n $$

where *C* = total chlorophyll concentration (mg/L), *FW* = tissue fresh weight (g), *V* = total extract volume (mL), and *n* = dilution factor.

### Statistical analysis

Data were analyzed with GraphPad Prism 10 (v10.1.2; GraphPad Software, Boston, MA, USA). Differences were determined by the Student’s *t*-test. Significance was accepted at *P* < 0.05.

## Results

### Isolation of high-efficiency feather-degrading microbial strains

**Fig. 1:**
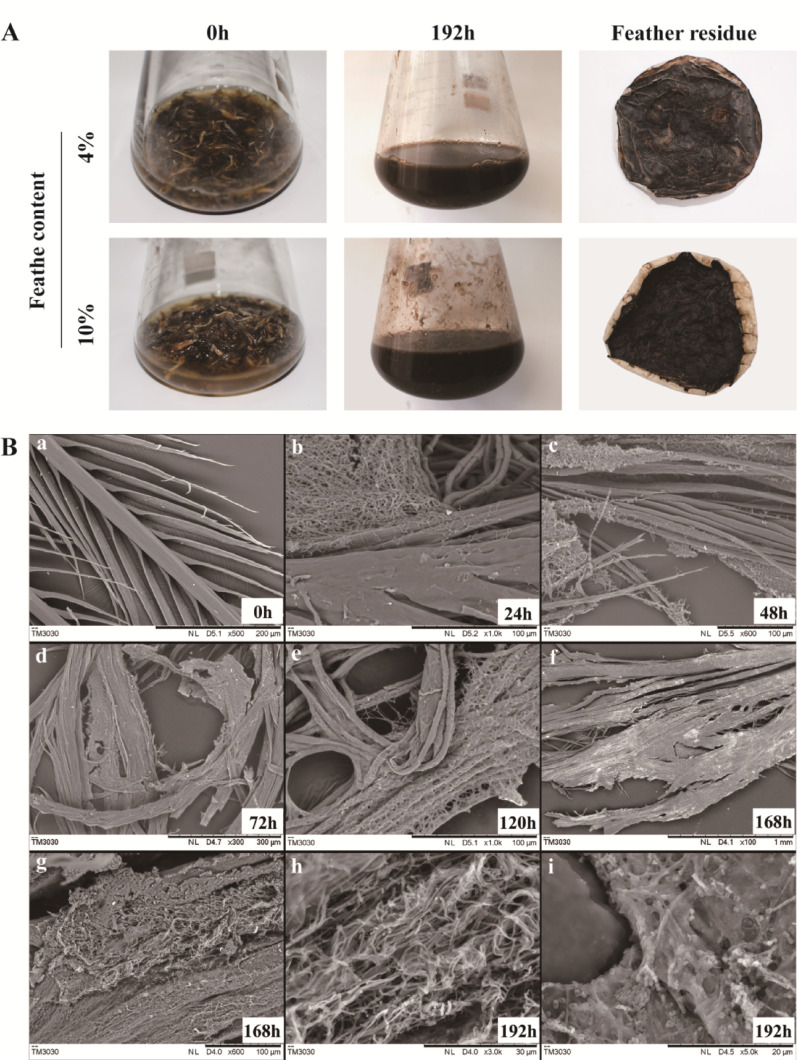
Efficient degradation and microstructural changes of chicken feathers by strain KK^T^. **A** degradation at 4.0% and 10.0% feather content **B** microstructural evolution of feathers during degradation (SEM)

A strain with superior feather-degrading efficiency, designated KK^T^, was isolated through enrichment culture and successive screening on feather meal agar. When cultivated with 4.0% (w/v) chicken feathers at 30 ℃ for 8 days, strain KK^T^ achieved a degradation percentage of 77.9 ± 0.9%. Notably, even at a high feather loading of 10.0% (w/v), it maintained a substantial degradation efficiency of 51.4 ± 0.9%. (Fig. 1A). Given its high performance, strain KK^T^ was selected for further characterization.

### SEM observations on chicken feather degradation process

The structural dynamics of chicken feathers during degradation by strain KK^T^ are presented in Fig. 1B. At 0 h (native state), feathers displayed intact architecture with well-organized barbs and barbules (Fig. 1Ba). Following 24 h incubation, mycelia extensively colonized feather surfaces, exhibiting radial growth patterns across barbules (Fig. 1Bb). By 48 h, mycelia tightly entangled and enveloped the barbules with incipient fracturing evident (Fig. 1Bc). Structural disintegration progressed rapidly between 72 and 168 h: rachises and barbules underwent extensive fragmentation while proliferating mycelia concurrently formed honeycomb networks that fully encapsulated feather debris (Fig. 1Bd-Bg). At 192 h (terminal phase), complete keratin breakdown was observed, accompanied by predominant sporulation (Fig. 1Bh-Bi).

### Phylogenetic analysis of strain KK^T^ based on 16S rRNA gene sequence

**Fig. 2:**
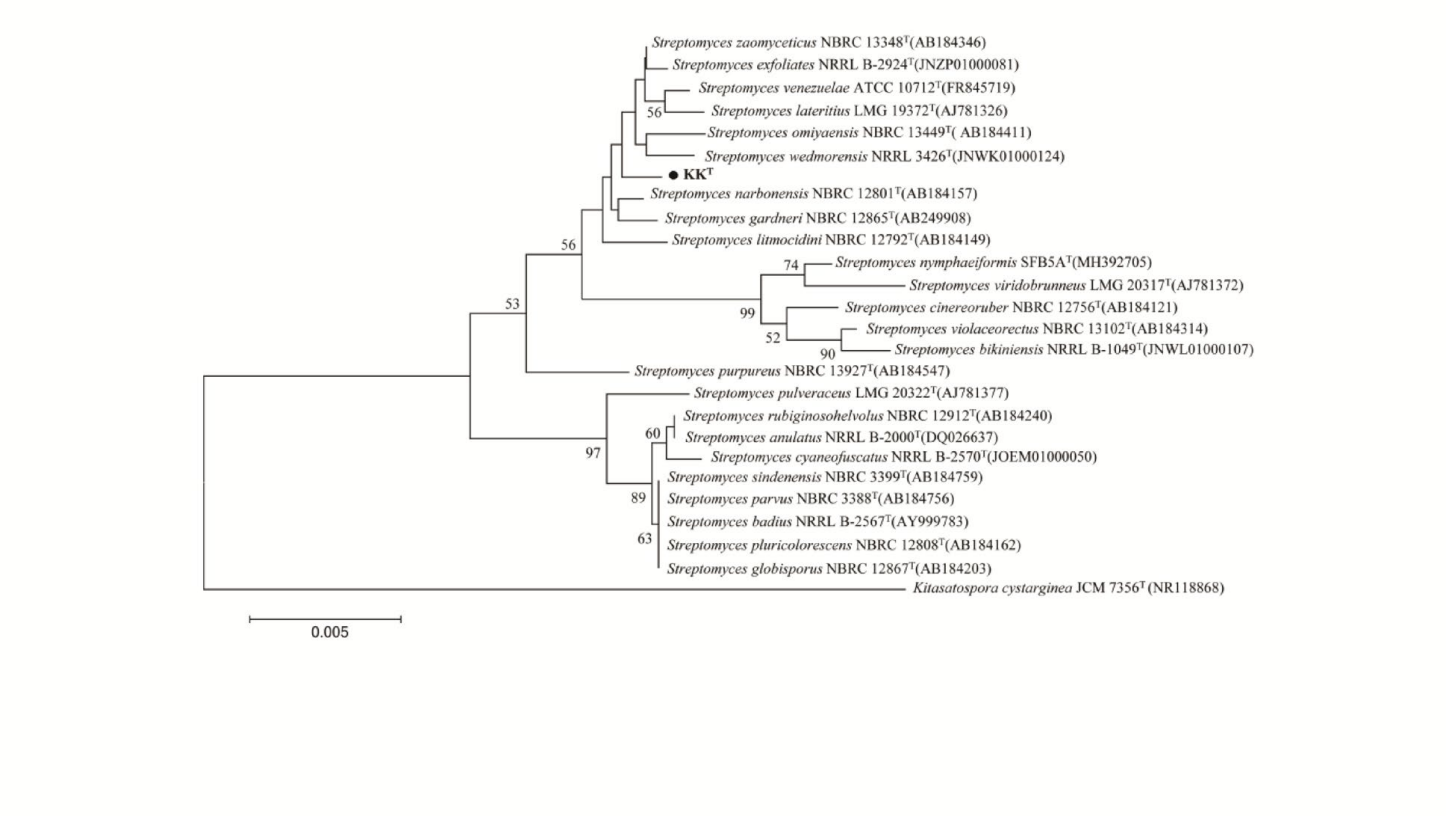
Neighbour-joining phylogenetic tree based on 16S rDNA sequences from strain KK^T^ and its closely related type strains. Only bootstrap values above 50% (based on 1000 replications) are indicated. The scale bar indicates 0.005 substitutions per nucleotide position. Strain *Kitasatospora cystarginea* JCM 7356^T^ (NR118868) was used as the out-group

The 16S rRNA gene sequence of strain KK^T^ (1,516 bp) was deposited in GenBank under Accession No. OR539445.1. Pairwise alignment via EzBioCloud revealed highest sequence similarity to *Streptomyces zaomyceticus* NBRC 13348^T^ (99.79%), followed by *S. exfoliatus* NRRL B-2924^T^ (99.72%), *S. narbonensis* NBRC 12801^T^ (99.72%), and *S. venezuelae* ATCC 10712^T^ (99.65%). Phylogenetic analysis using neighbor-joining (NJ) and maximum-likelihood (ML) methods (Fig. 2; Supplementary Figure S1) indicated that strain KK^T^ formed a distinct clade, clustering with type strains of *S. zaomyceticus*, *S. exfoliatus*, *S. venezuelae*, *S. narbonensis*, *S. gardneri*, *S. wedmorensis*, and *S. cinereoruber*. Together, these findings confirm strain KK^T^ as a member of the genus *Streptomyces*.

### Phenotypic characteristics of strain KK^T^

**Fig. 3:**
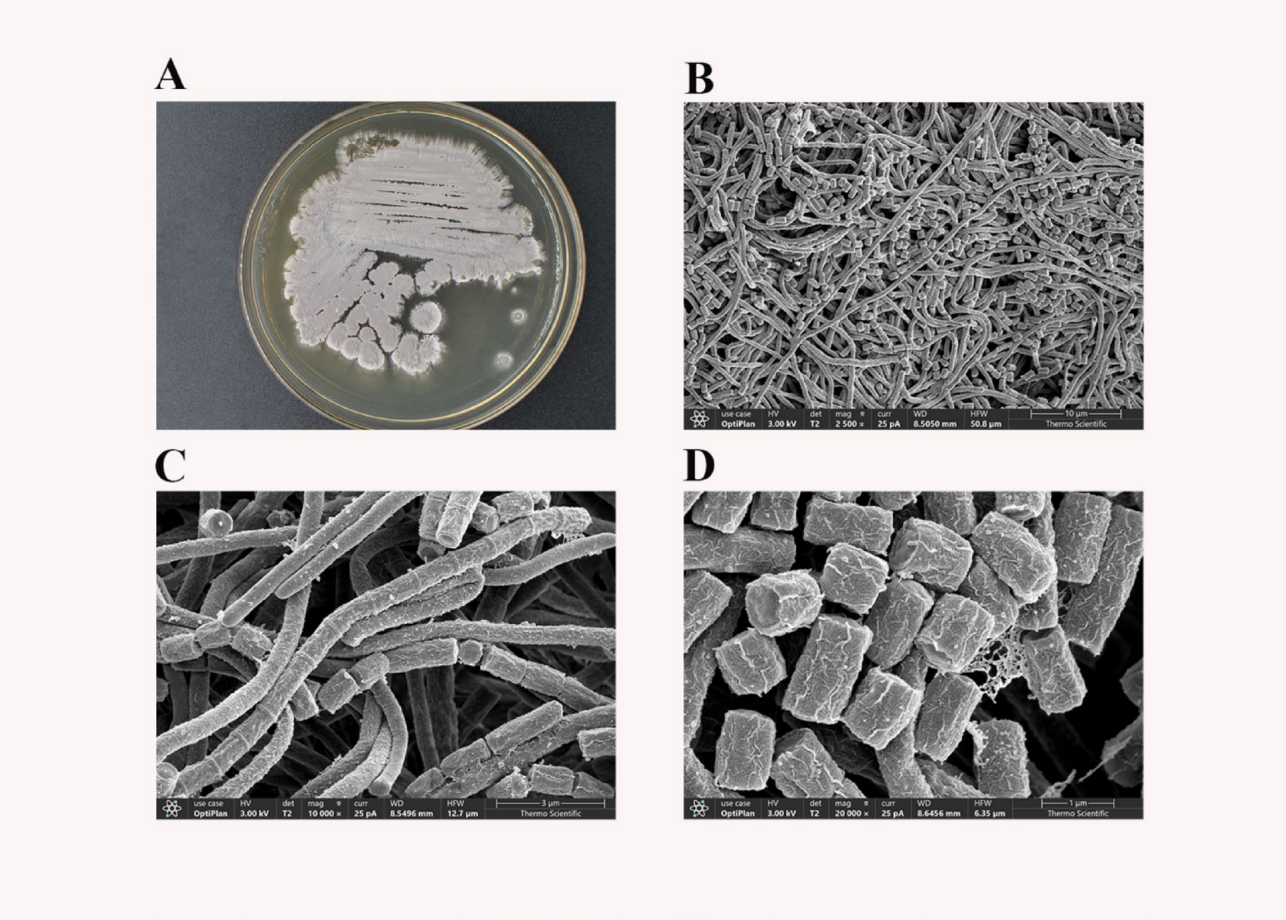
Morphological characteristics of strain KK^T^. **A** morphological features of strain KK^T^ on ISP2 (**B**–**D**) scanning electron micrograph of strain showing spore chains and spore surface

Strain KK^T^ displayed vigorous growth on ten media (ISP1-7, NA, CZ, and PDA; Supplementary Figure S2), exhibiting typical *Streptomyces* morphology. The aerial mass showed coloration ranging from white to gray, while substrate mycelia were medium-dependent, appearing in white or yellow color series, specifically white, ivory, beige, ochre-yellow or gray-beige. Aerial hyphae formed long fascicles that differentiated into rectiflexible spore chains containing over 20 spores. Spores were cylindrical (0.6 × [0.7–1.5] µm), with an ornamented-reticulate surface (Fig. 3). Melanoid pigments were produced on ISP1, ISP2, ISP6, ISP7, and PDA, while a diffusible pink pigment was observed on ISP3. Optimal growth occurred at pH 7.0 (growth range 6.0–9.0), and 30 ℃ (growth range 10–43 °C), with tolerated NaCl concentrations up to 5.0% (w/v). Strain KK^T^ grew on glucose, mannose, xylose, maltose, galactose, and fructose as sole carbon sources. Lactose, ribose, and sorbitol utilization yielded ambiguous growth responses. Among nitrogen sources, leucine, valine, glutamine, asparagine, threonine, proline, alanine, serine, and glycine supported growth as sole nitrogen sources, whereas methionine utilization remained unconfirmed (Table 1).

**Table 1:** Comparision of phenotypic characteristics between KK^T^ and its closest type strains

Characteristics	1	2	3	4 ^a, c, d^
Morphological characteristics				
Aerial mycelium	White or gray series	White or yellow series	White series	Red or gray series
Substrate mycelium	White or yellow series	White or yellow series	White series	Red or yellow series
Soluble pigment	Melanoid and pink	Melanoid	None	Melanoid
Spore surface	Rugose	Smooth ^*a*^	Smooth ^*a*^	Smooth
Ecological, physiological and biochemical characteristics				
Growth temperature	10–43 ℃	10–38 ℃	5–42 ℃	10–45 ℃
pH range for growth	6.0–9.0	6.0–9.0	6.0–9.0	6.0–9.0
NaCl tolerance (w/v, %)	0–5.0	0–4.5	0–5.5	0–5.0
Catalase	+	+	+	+
Oxidase	+	-	-	-
Nitrate reduction	+	+	+	+
Production of H_2_S	+	-	+	+
Peptonization of milk	+	+	+	ND
Aesculin hydrolysis	+	+	+	ND
Urease production	+	+	+	-
Gelatin degradation	+	+	+	+
Starch degradation	+	+	-	ND
Tween 80 degradation	+	+	+	+
API ZYM test				
Alkaline phosphatase	+	+	+	+
Esterase(C4)	+	+	+	+
Esterase lipase(C8)	+	+	+	+
Lipase(C14)	-	-	-	+
Leucine arylamidase	+	+	+	+
Valine arylaminase	+	+	+	+
Cystine arylaminase	+	+	+	+
Trypsin	+	+	+	+
Chymotrypsin	+	+	+	+
Acid phosphatase	+	+	+	+
Naphthol-AS-BI-phosphohydrolase	+	+	+	+
α-galactosidase	-	-	+	-
β-galactosidase	+	+	+	+
β-glucuronidase	-	-	-	-
α-glucosidase	+	+	+	+
β-glucosidase	+	+	+	+
N-acetyl-beta-glucosaminidase	+	+	+	+
α-Mannosidase	-	-	-	-
α-Fucosidase	+	-	-	-
Sole carbon source utilization				
D-raffinose	-	-	-	+
D-glucose	++	++	++	+
D-mannitol	-	-	-	+
D-mannose	+	-	++	+
L-arabinose	-	+	+	+
Lactose	±	±	++	ND
D-ribose	±	-	+	+
D-xylose	+	+	++	+
D-sorbitol	±	-	-	ND
Sucrose	-	-	-	-
Rhamnose	-	-	+	-
Maltose	++	++	++	ND
Inositol	-	-	±	+
D-galactose	++	+	++	+
D-fructose	+	-	++	±
Sole nitrogen source utilization				
L-glutamic acid	-	-	-	+
L-leucine	+	+	+	ND
Creatine	-	-	-	ND
L-valine	+	+	+	ND
L-glutamine	+	+	+	ND
L-aspartic acid	-	-	-	ND
L-asparagine	+	+	+	ND
L-threonine	+	+	+	ND
L-methionine	±	±	±	+
L-proline	+	+	+	ND
L-alanine	+	+	+	+
L-serine	+	+	+	+
L-glycine	+	+	+	+
Chemotaxonomic characteristics				
Major fatty acids (percent > 10%)	anteiso-C_15:0_, iso-C_16:0_, anteiso-C_17:0_, iso-C_15:0_	ND	anteiso-C_15:0_, anteiso-C_17:0_, iso-C_15:0_, C_16:0_^*c*^	anteiso-C_15:0_, iso-C_16:0_, iso-C_14:0_
Major phospholipid	DPG, PE, PI, PL, APL	DPG, PE ^*b*^	ND	ND
Major menaquinone	MK-10(H4), MK-9(H8)	ND	ND	MK-9(H6), MK-9(H8)

Strains: 1, *Streptomyces shaoguanensis* KK^T^; 2, *S. zaomyceticus* CGMCC4.1953^T^; 3, *S. exfoliatus* GDMCC4.124^T^; 4, *S. cinereoruber* subsp. *cinereoruber* DSM 41512^T^. ^*a*^, data from ref. [65]; ^*b*^, ref. [66]; ^*c*^, ref. [67]; ^*d*^, ref. [68]

++ strongly positive utilization; + positive utilization; ± utilization doubtful; - utilization negative

Abbreviations: PE, phosphatidylethanolamine; DPG, diphosphatidylglycerol; PL, unidentified phospholipid; PI, phosphatidylinositol; APL, unidentified aminophospholipid

Chemotaxonomic analysis of strain KK^T^ revealed LL-diaminopimelic acid (DAP) in the cell wall, with galactose and ribose in whole-cell hydrolysates (Supplementary Figure S3). Polar lipids included diphosphatidylglycerol (DPG), phosphatidylethanolamine (PE), phosphatidylinositol (PI), unidentified phospholipid (PL), and unidentified aminophospholipid (APL) (Supplementary Figure S4). Menaquinones comprised MK-10 (H₄) (66.58%) and MK-9 (H₈) (33.42%). Major fatty acids (> 10% each) were anteiso-C₁₅:_0_ (22.33%), iso-C₁₆:_0_ (15.84%), anteiso-C₁₇:_0_ (11.23%), and iso-C₁₅:_0_ (11.11%) (Table 1 and Supplementary Table S1). The phenotypic (morphological, physiological, and biochemical) and chemotaxonomic profiles of strain KK^T^ were consistent with those of members of the genus *Streptomyces* [68]. However, phenotypic differences were evident when compared to its closest phylogenetic relatives, particularly in key morphological and physiological traits (e.g., aerial mycelium color, substrate mycelium color, spore surface ornamentation, and growth temperature range; Table 1) [69–72].

### Phylogenomic analysis of strain KK^T^

**Fig. 4:**
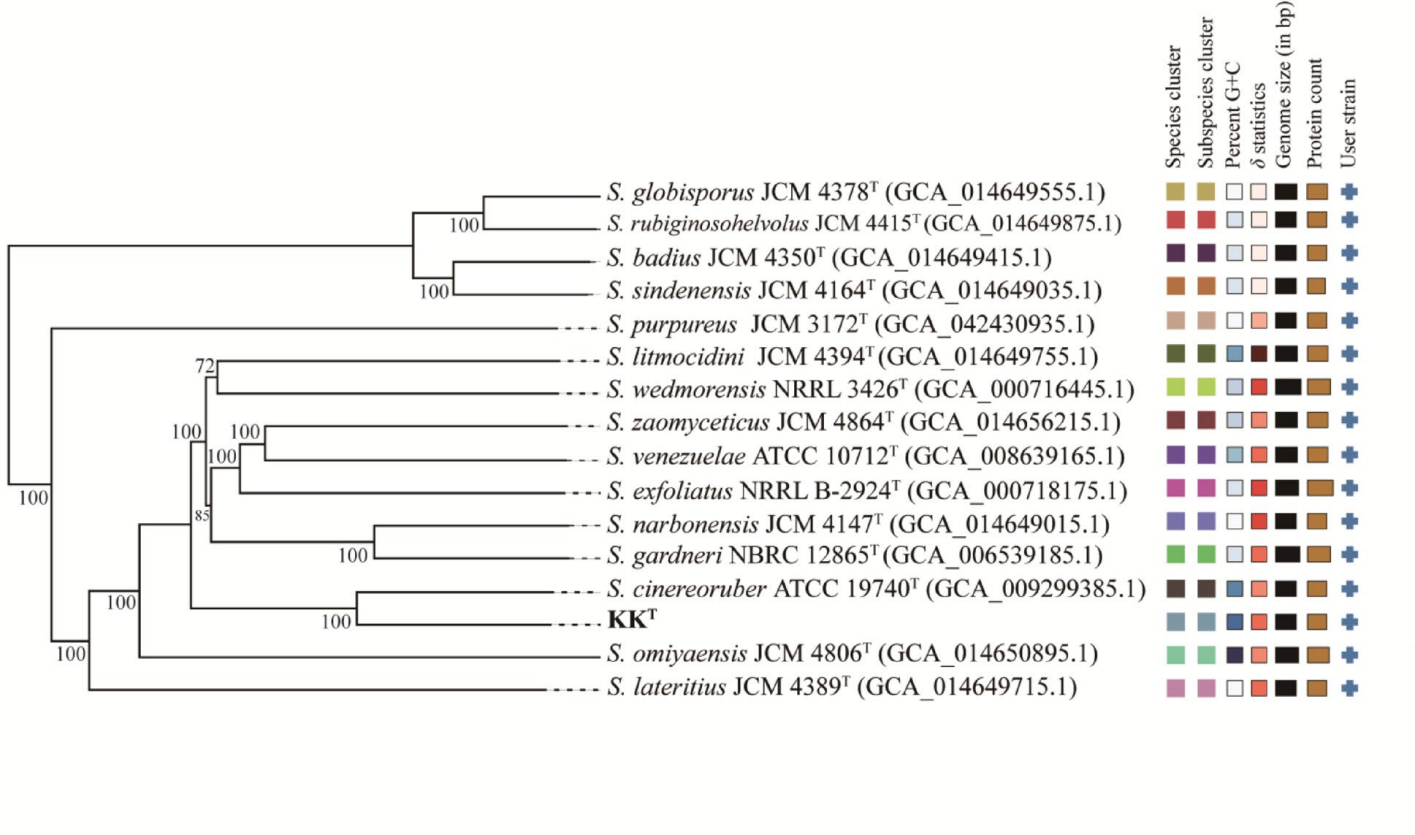
Phylogenomic tree of strain KK^T^ and closely related type strains constructed using TYGS. Tree inferred with FASTME 2.1.6.1 based on GBDP distance matrices computed from genome sequences, with branch lengths scaled by the GBDP formula d_5_. GBDP pseudo-bootstrap support values (> 60%) from 100 replications are shown above branches, with an overall average of 96.8%

Strain KK^T^ showed the highest ANI (92.99%) and dDDH (51.20%) values against *S. cinereoruber* ATCC 19740^T^. For all other closely related *Streptomyces* type strains (selected based on ≥ 98.7% 16S rRNA gene sequence similarity, Supplementary Table S2), the maximum obtained values were only 87.49% (ANI) and 34.70% (dDDH). Notably, all pairwise comparisons revealed values well below the accepted species delineation thresholds (ANI < 95–96%; dDDH < 70%) [73–75]. These data demonstrated that strain KK^T^ represented a novel species within the genus *Streptomyces*. Phylogenomic analysis clustered strain KK^T^ and *S. cinereoruber* ATCC 19740^T^ into a well-supported clade (100% bootstrap; Fig. 4), which was further nested within a larger cluster (72–99% bootstrap) containing *S. zaomyceticus* JCM4864^T^, *S. exfoliatus* NRRL B-2924^T^, *S. venezuelae* ATCC 10712^T^, *S. narbonensis* JCM 4147^T^, *S. gardneri* NBRC 12865^T^, *S. litmocidini* JCM4394^T^ and *S. wedmorensis* NRRL 3426^T^. In contrast, 16S rRNA gene phylogeny positioned strain KK^T^ within a weakly supported clade (56% bootstrap) adjacent to *S. wedmorensis* NRRL 3426^T^ and *S. zaomyceticus* NBRC 13348^T^, whereas *S. cinereoruber* NBRC 12756^T^ formed a distinct, highly supported cluster (99% bootstrap) with *S. viridobrunneus* LMG 20317^T^, highlighting the limitations of single-gene markers in resolving species boundaries. Notably, this weak phylogenetic resolution aligns with the high 16S rRNA gene sequence similarity (≥ 98.7%) between strain KK^T^ and its adjacent clade members, yet contrasts sharply with their low genomic relatedness as measured by ANI and dDDH. This discrepancy is not uncommon in speciose genera like *Streptomyces* (comprising 1,357 species and 83 subspecies at the time of writing; data were from LPSN, https://lpsn.dsmz.de). The conserved nature of the 16S rRNA gene, coupled with potential horizontal gene transfer, often limits its discriminatory power [76, 77]. Genome-wide analyses (ANI and dDDH), by integrating information from thousands of loci [55], provide a more robust assessment of genomic relatedness, as evidenced by the current data.

In conclusion, comprehensive polyphasic taxonomic analyses including phenotypic, chemotaxonomic, and genomic data support the recognition of strain KK^T^ as a novel species of the genus *Streptomyces*, for which the name *Streptomyces shaoguanensis* sp. nov. is proposed.

### Genomic features and functional annotation of *Streptomyces shaoguanensis* KK^T^

Genome sequencing of strain KK^T^ yielded 11,280,580 Illumina and 3,403,037 PacBio raw reads. After quality control, 10,988,770 high-quality Illumina reads (Q30: 90.44%) and 2,591,652 PacBio reads (mean length: 2,965 bp; N50: 5,478 bp) were obtained. The hybrid assembly achieved 99.86% genome coverage, producing a complete but non-circularized genome of 7,843,677 bp (73.21% GC content) comprised of 5 contigs (N50: 7,529,971 bp; mean length: 1,568,760 bp; GenBank accession: JASJAK000000000) (Supplementary Figure S5). In addition to the chromosome, contig00003 (63,878 bp) was identified as a complete plasmid. Plasmid identification was performed using MOB-suite (v3.1.9) [78], Plasmer (v0.1) [79], and PLASMe (v1.1) [80]. This conclusion was supported by multiple lines of evidence: the presence of key plasmid markers, including a replication origin (rep_cluster_43) and an MOBF-family relaxase; a high Plasmer classification probability (0.874); and a high-confidence match (Score = 0.999) to plasmid pSLE2 from *S. leeuwenhoekii* in the PLASMe database. Prokka-based annotation predicted 6,779 protein-coding sequences (CDSs), 18 rRNA genes, and 89 tRNA genes, with details provided in Supplementary Table S3.

COG annotation assigned functional categories to 4,106 genes (60.6% of CDSs), with metabolic processes representing the predominant category (1,869 genes, 45.5% of COG-annotated genes), followed by cellular processes and signaling (905, 22.1%), information storage and processing (883, 21.5%), and poorly characterized (449, 10.9%). Subclassification into 23 functional groups revealed significant representation in: transcription (503), general function prediction only (340), amino acid transport and metabolism (320), carbohydrate transport and metabolism (307), lipid transport and metabolism (306), signal transduction mechanisms (304), and energy production and conversion (270) (Supplementary Figure S6).

GO annotation via InterProScan assigned functional terms to 4,022 genes. Molecular function constituted the largest category (3,644 genes), with catalytic activity (2,128 genes) and binding (1,828 genes) as the most abundant subclasses. Biological processes and cellular components included 2,671 and 1,544 genes respectively, dominated by cellular process (2,078 genes) and intracellular anatomical structure (665 genes) (Supplementary Figure S7).

KEGG pathway annotation assigned K numbers (KEGG Orthology identifiers) to 2,291 genes (34.3% of CDSs), of which 1,803 genes mapped to specific metabolic pathways. The most enriched pathway was ko01100 (Metabolic pathways), dominated by amino acid metabolism (262 genes), carbohydrate metabolism (237), and metabolism of cofactors and vitamins (200) (Supplementary Figure S8).

Genome mining via antiSMASH 7.1.0 identified 26 putative biosynthetic gene clusters (BGCs) in strain KK^T^, with clusters 1–25 localized on Contig 1 and cluster 26 on Contig 2, spanning seven major metabolic categories (Supplementary Table S4). Twelve BGCs exhibited ≥ 60% similarity to known reference clusters, including four terpenoid derivatives (geosmin, isorenieratene, 2-methylisoborneol, hopene), three hybrid types, and single clusters encoding non-ribosomal peptide synthetase (NRPS), ectoine, siderophore, type I polyketide synthase (T1PKS), and lassopeptide. The remaining 14 BGCs exhibited less than 30% similarity to any characterized clusters. Their predicted classes comprised two clusters each of terpenoid, hybrid, NRPS, and melanin types; and one cluster each of siderophore, ribosomally synthesized and post-translationally modified peptide (RiPP), hydrogen cyanide, butyrolactone, and N-acetyl-para-aminophenol analog (NAPAA). Notably, one additional cluster annotated as RiPP-like showed no significant homology to any known gene clusters in current databases.

### Biochemical signatures of feather degradation mediated by strain KK^T^

**Fig. 5:**
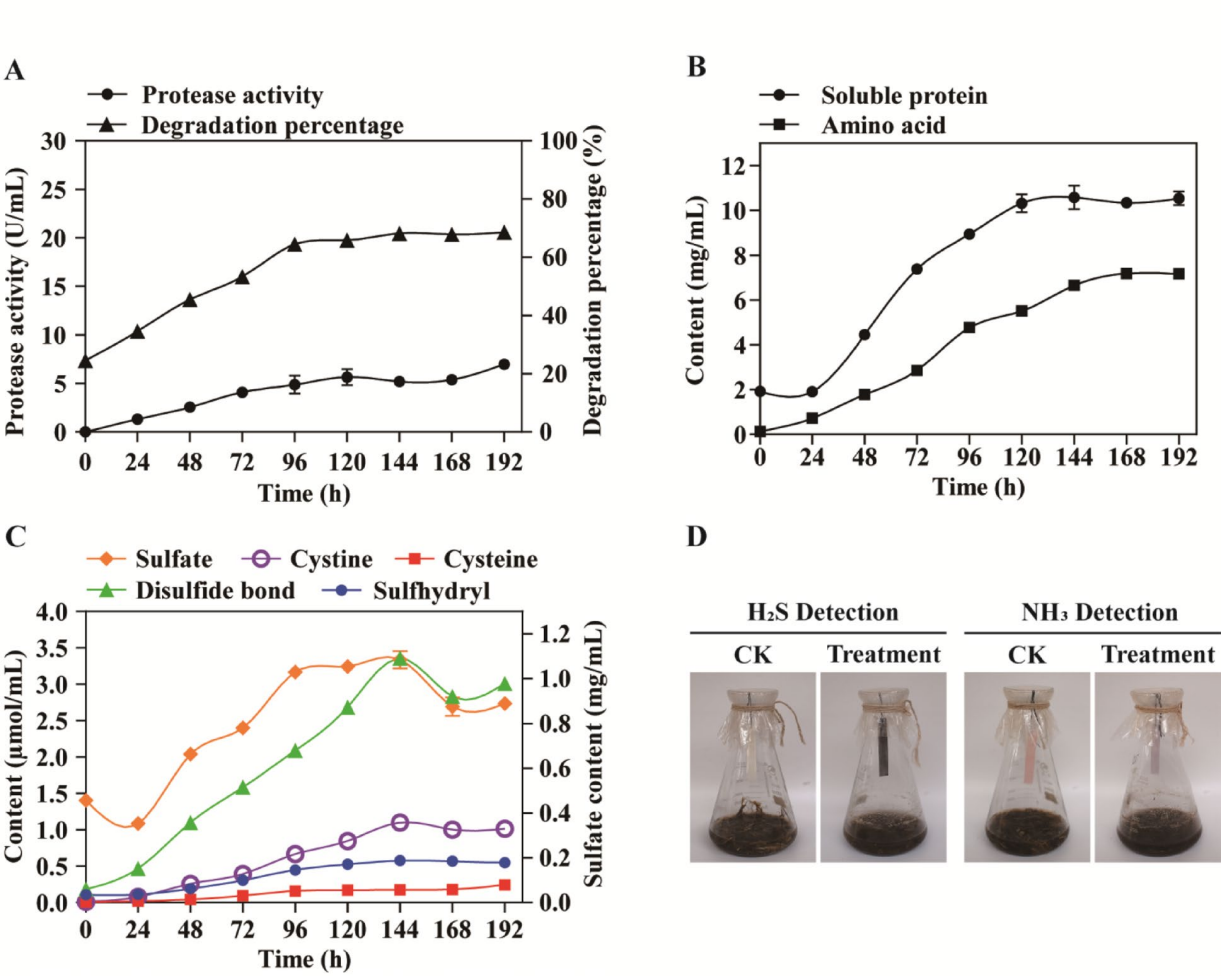
Dynamic bioconversion profiles of feather hydrolysate by strain KK^T^. **A** Time-course feather degradation efficiency and proteolytic activity **B** Temporal dynamics of nitrogenous metabolites: free amino acids vs. soluble proteins **C** Sulfur-containing compounds accumulation during keratinolysis **D** Gaseous metabolite release (H₂S/NH₃) monitored by colorimetric assays

The biochemical profile of feather degradation by strain KK^T^ are presented in Fig. 5. Soluble proteins (1,918.50 ± 30.00 µg/mL), amino acids (135.28 ± 1.96 µg/mL), disulfide bonds (0.18 µmol/mL), thiol groups (0.11 µmol/mL), and sulfate (456.37 ± 21.10 µg/mL) were detected in the supernatant at 0 h (immediately after autoclaving), with concurrent release of volatile H₂S (qualitatively detected).

Following 24 h of fermentation, the sulfate concentration decreased to 352.96 µg/mL, while soluble protein content exhibited a slight reduction, reaching 1,908.50 µg/mL. No significant changes were observed in the levels of thiol groups, cysteine, or cystine (Fig. 5B and C).

Feather degradation efficiency increased significantly during the 24–120 h fermentation period, reaching 65.9 ± 1.5% at 120 h. This was accompanied by concurrent increases in extracellular protease activity, soluble protein concentration, total amino acid content, and levels of sulfur-containing compounds (including sulfate, cystine, thiol groups, and cysteine) (Fig. 5A and B, and 5C).

During the later fermentation phase (120–192 h), feather degradation efficiency stabilized, peaking at 68.6 ± 0.8% by 192 h, while protease activity reached its maximum of 6.99 ± 0.52 U/mL and remained constant thereafter. Soluble protein concentration peaked at 10,585.00 ± 526.00 µg/mL (144 h), followed by total amino acids at 7,188.00 ± 42.80 µg/mL (168 h), both subsequently maintained steady-state levels. Multiple sulfur-containing metabolites, including free thiol groups (0.58 ± 0.01 µmol/mL), cystine (1.10 ± 0.02 µmol/mL), sulfate (1,085.16 ± 68.29 µg/mL), and disulfide bonds (3.36 ± 0.03 µmol/mL), reached peak concentrations at 144 h. Beyond 168 h, sulfate and disulfide bonds decreased significantly, stabilizing at 875.56 ± 71.30 µg/mL and 2.83 ± 0.04 µmol/mL, respectively, whereas cystine and sulfhydryl showed modest reduction. The cysteine level remained consistent at 0.24 µmol/mL after 120 h (Fig. 5A and B, and 5C). Notably, sulfite was detectable exclusively between 96 and 144 h at trace-level concentrations (2.0–3.2 mg/L).

The timing of gas release showed that lead acetate test paper fully darkened at 17 h, confirming H₂S production. Red litmus paper began turning blue at 30 h and was fully blue by 48 h, indicating NH₃ production. Both gases were produced continuously until 120 h (Fig. 5D). As a result of this cumulative NH₃ release, the pH of the fermentation broth elevated significantly, from 7.48 ± 0.12 at 0 h to a stabilized 9.12 ± 0.08 after 120 h.

### Quality assessment of RNA-Seq data

Illumina high-throughput sequencing of the 12 samples (three experimental groups and one control, with three biological replicates per group) generated between 12.5 and 19.6 million raw reads per sample. The high quality of the raw data was evidenced by Q20 and Q30 values exceeding 97.7% and 93.4%, respectively. After quality control, an average of 97.5% of the reads (ranging from 96.0% to 98.1%) were retained as high-quality clean data. Mapping these reads to the reference genome of strain KK^T^ achieved a total mapping rate between 97.4% and 99.2%, demonstrating the dataset’s suitability for subsequent analysis. Detailed statistics for each sample are provided in Supplementary Table S5.

### Expression profiling of keratin-degrading genes in strain KK^T^

Integrated annotation leveraging NR, RefSeq, UniProt, and MEROPS databases identified 208 putative protease-encoding genes within the strain KK^T^ genome. Among these, 189 were classified into 47 protease families distributed across 30 clans (Supplementary Table S6 and S7). Applying a significance threshold (log₂FC ≥ 1.5, *P* < 0.05), 61 protease genes exhibited significant upregulation during keratin degradation. At 24 h versus 0 h, 29 protease genes showed pronounced upregulation, predominantly encoding serine proteases and metalloproteases (Supplementary Table S8A). This cohort of 21 secreted protease-encoding genes included six putative keratinase genes: S08 family members *KK_01510* (Prokka gene ID; 60.57-fold), *KK_01648* (15.60-fold), *KK_02372* (15.33-fold), *KK_05679* (7.79-fold), *KK_06063* (3.18-fold), and M04 family member *KK_04748* (10.29-fold). Notably, the unclassified papain-like cysteine protease family protein *KK_01746* and M06 metalloprotease *KK_02604* demonstrated striking upregulation (193.44-fold and 21.80-fold, respectively), while ATP-dependent Clp protease genes *KK_02348* and *KK_02349* peaked at 48 h (14.25-fold and 4.71-fold versus 0 h). Additionally, seven protease genes exhibited upregulation at 48 h, and thirty were upregulated at 96 h, among which five (*KK_04748*, *KK_03591*, *KK_05679*, *KK_03974*, and *KK_05093*) showed upregulation initiating at 24 h (Supplementary Table S8B and S8C, respectively).

Genes associated with disulfide bond reduction [81, 82] exhibited differential expression profiles versus 0 h (Supplementary Table S9A). Thioredoxin genes *trxA* and *trxC* displayed 3.47-fold and 2.21-fold upregulation at 48 h. Thioredoxin reductase genes *trxB_3* and *trxB_2* showed 2.13-fold upregulation at 24 h and 7.85-fold upregulation at 96 h, respectively. Additionally, alkyl hydroperoxide reductase genes *ahpC* and *ahpD* were upregulated 12.28-fold and 12.24-fold at 24 h. Peak expression of peptide methionine sulfoxide reductase genes *msrA* and *msrB* occurred at 48 h with 1.94-fold and 2.27-fold upregulation. Ribonucleotide reductase genes *nrdB* and *KK_04579* were upregulated at 24 h (5.05-fold and 4.63-fold, respectively). Additionally, *nrdJ* was upregulated 3.44-fold at 48 h. Meanwhile, the dihydrolipoamide dehydrogenase genes *pdhD* and *lpdC* were upregulated 2.56-fold at 24 h and 4.38-fold at 48 h.

Genomic analysis of strain KK^T^ revealed a complete sulfate assimilation pathway, as shown in Supplementary Table S10A. Genes encoding key enzymes—including sulfate adenylyltransferase (*cysD*, *cysNC*; EC 2.7.7.4), adenylyl-sulfate kinase (*cysC*; EC 2.7.1.25), phosphoadenylyl-sulfate reductase (*cysH*; EC 1.8.4.8), and sulfite reductase (*sir*; EC 1.8.7.1)—were significantly downregulated during 24–48 h versus 0 h but showed variable upregulation at 96 h. In contrast, cysteine synthase gene *cysM* displayed no significant differential expression. The extracellular sulfite oxidase gene *suox* was downregulated at 24–48 h yet exhibited 4.71-fold upregulation at 96 h versus 0 h. Among seven sulfate transporter genes, only *ybaR* maintained consistent upregulation during 24–96 h, while among five sulfite efflux transporter genes, *KK_00432* showed sustained upregulation during this period.

Cysteine is oxidatively deaminated to sulfite by cysteine dioxygenase (CdoI) and aspartate aminotransferase (AspC). In strain KK^T^, two CdoI genes (*KK_05030* and *KK_02709*; Prokka IDs) exhibited basal expression during 0–48 h, with FPKM values ranging from 0 to 6, but surged at 96 h with 21.65-fold and 6.36-fold upregulation versus 0 h. Conversely, the AspC gene *KK_04063* maintained high expression (FPKM > 350) at 24 h and 48 h, upregulated 3.33-fold and 2.31-fold versus 0 h, with the other, *KK_01765*, showing a 10.30-fold upregulation at 48 h. Cysteine desulfhydrase (Cds1) catalyzes the desulfurization of cysteine, generating pyruvate, H₂S, and NH₃. Meanwhile, the cysteine desulfurase SufS, together with sulfur-carrier protein SufU, mobilizes sulfur from cysteine and transfers it to the SufBCD scaffold complex, where the sulfur is incorporated into Fe-S clusters for maturation of recipient apoproteins. In strain KK^T^, *cds1* was significantly upregulated at 24 h and 48 h by 2.92-fold and 2.23-fold, respectively, versus 0 h. Genes encoding Fe-S cluster assembly components (*sufS*, *sufU*, *sufB*, *sufC*, *sufD*) and Fe-S protein maturation accessory factor *yitW* all reached peak expression at 48 h, exhibiting 2.95-fold, 2.52-fold, 2.01-fold, 5.09-fold, 5.15-fold, and 3.05-fold upregulation versus 0 h, respectively (Supplementary Table S10B). While glutathione (GSH) biosynthesis from cysteine promotes keratin degradation through disulfide reductase activation [83], many actinomycetes utilize mycothiol (AcCysGlcN-Ins, MSH) as an alternative thiol cofactor [84]. Genomic analysis of strain KK^T^ revealed the absence of γ-glutamylcysteine synthetase—precluding GSH biosynthesis—but confirmed functional MSH biosynthesis operons (*mshA*, *mshB*, *mshC*, *mshD*). These genes exhibited temporal upregulation at distinct degradation stages (Supplementary Table S10B).

Beyond the aforementioned genes, additional genetic determinants displayed significant transcriptional responses. Genes encoding peptide and amino acid transporters were markedly upregulated, including the oligopeptide transporter gene *oppC*, dipeptide transporter gene *dppA*, di-/tripeptide transporter gene *dtpT*, peptide-binding protein genes *oppA*, *oppD*, and *ddpA* (Supplementary Table S9B), as well as branched-chain amino acid transporter genes (*livF* and *livH*) and amino acid permease genes (*KK_00520* and *yhdG*) (Supplementary Table S9C). Concurrently, the expression of several stress-response genes was upregulated, most markedly at 48 h versus 0 h in *sodF* (32.74-fold), *fusA* (12.28-fold), *tuf* (7.02-fold), *hsp18* (5.85-fold), and *groL2* (2.57-fold) (Supplementary Table S9D). Furthermore, genes encoding enzymes involved in core metabolic pathways—the tricarboxylic acid (TCA) cycle, gluconeogenesis, and fatty acid synthesis—were significantly upregulated (Supplementary Tables S10C-S10E).

### Growth response of *Brassica rapa* subsp. *chinensis* (Pak Choi) to chicken feather hydrolysate

**Fig. 6:**
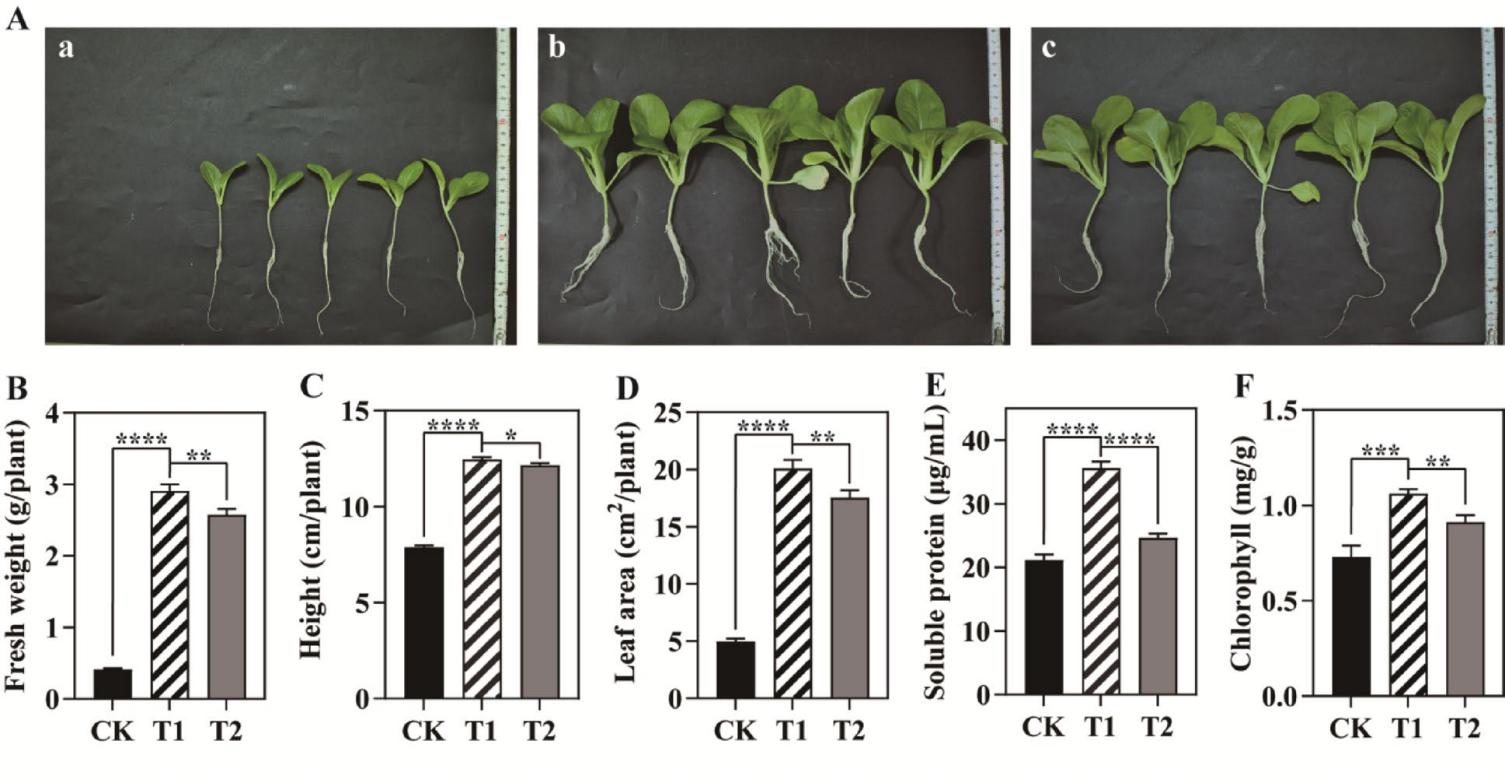
Phenotypic response and growth parameters of Pak choi to different fertilizer regimes. **A** Plant phenotypes. **B** Fresh weight. **C** Plant height. **D** Leaf area. **E** Soluble protein content. **F** Chlorophyll content. Treatments: CK (Control), T1 (Chicken feather degradation liquid), T2 (Commercial amino acid fertilizer). Significance markers: “****” (*P* < 0.0001), “***” (*P* < 0.001), “**” (*P* < 0.01), “*” (*P* < 0.05). Bar graphs show mean ± SD (*n* = 25)

Free amino acid profiling revealed that 10.0% CFH contains multiple amino acids but has significantly lower concentrations of key plant-growth-promoting amino acids than commercial fertilizer—specifically glycine (29-fold lower), glutamate (18-fold lower), and undetectable serine/aspartate levels (full data: Supplementary Table S11). To enable comparative efficacy assessment, the total free amino acid content in CFH was doubled relative to the commercial fertilizer (2:1 ratio), mitigating critical amino acid concentration disparities. Figure 6 presents the effects of three fertilization regimens on Pak Choi. Both CFH (Fig. 6Ab) and commercial fertilizer (Fig. 6Ac) significantly enhanced plant morphology compared to the control (Fig. 6Aa), resulting in predominantly eight-leaf plants with robust root systems, whereas control plants had six leaves and underdeveloped roots. CFH-treated plants exhibited substantial increases in growth metrics for fresh weight (+ 604.2%), plant height (+ 57.9%), leaf area (+ 303.7%), soluble protein content (+ 68.2%), and chlorophyll levels (+ 45.3%) relative to controls (all *P* < 0.001). Notably, CFH treatment generated 44.4% higher soluble protein content than commercial fertilizer (*P* < 0.001). CFH also outperformed commercial fertilizer in fresh weight (+ 13.1%), leaf area (+ 14.4%), and chlorophyll level (+ 16.3%) (all *P* < 0.01), with plant height significantly greater by 2.5% (*P* < 0.05) (Fig. 6B and F).

## Discussion

### Biotechnological potential of novel strain KK^T^: keratin degradation and secondary metabolite production

*Bacillus* spp. are widely studied for feather degradation due to their efficient keratinase secretion, and *B. licheniformis* is recognized as a particularly effective species [85]. However, most isolates only achieve high degradation percentages at low feather concentrations (≤ 1.0% w/v). For instance, *Bacillus* sp. A5.3 degraded 70.0% at 0.75% w/v after 7 d [86], while *B. subtilis* ALICA and *B. cereus* C3752 achieved 86.0 ± 1.5% (5 d) and 90.5% (30 h) at 1.0% w/v, respectively [19, 87]. Although certain *B. licheniformis* strains retain degradation activity at elevated substrate concentrations, their efficiency remains limited—e.g., BBE11-1 achieved only 35.4% degradation at 5.0% w/v after 96 h [2]. While process optimization enabled strain ZSZ6 to attain 70.0% degradation at 5.0% w/v over 9 days (the highest reported efficiency for *Bacillus* under enhanced conditions) [88], our isolated wild-type strain KK^T^ displayed an exceptional innate capacity. Without any optimization and in sulfur-supplemented fermentation medium, strain KK^T^ achieved efficiencies of 41.4 ± 0.8% at 4.0% w/v after 96 h and 77.9 ± 0.9% after 192 h, surpassing both the unoptimized BBE11-1 and the fermentation-optimized ZSZ6. Remarkably, strain KK^T^ maintained a 51.4 ± 0.9% efficiency even at 10.0% w/v, establishing it as a uniquely effective wild-type strain capable of high-efficiency degradation at concentrations ≥ 5.0% w/v.

Amino acids regulate plant growth, quality, and stress resistance: glycine elevates biomass and nutrition [89, 90]; glutamate and aspartate facilitate nutrient assimilation [91]; proline enhances multi-stress tolerance (e.g., drought, salt, heavy metals) [92, 93]; serine and valine mitigate thermal stress; and alanine stimulates chlorophyll biosynthesis [91]. Microbial feather degradation generates nitrogen-rich hydrolysates (soluble proteins, peptides, amino acids) that offer sustained bioavailability, creating a valuable biofertilizer [7, 94]. However, the compositional profile of these hydrolysates is strain-dependent. For instance, *B. cereus* IIPK35 produces hydrolysates dominated by threonine and serine [95]; *B. velezensis* NA16 yields a phenylalanine-rich profile [82]; while *Ectobacillus* sp. JY-23 generates a composition abundant in glycine and proline [54]. The CFH produced by strain KK^T^ exhibited a valine- and leucine-dominated profile, a composition known to enhance stress resilience and biomass when foliar-applied, as demonstrated in *Camelina sativa* (improved stress tolerance, plant height, and fresh weight) [96]. In Pak Choi trials, the CFH outperformed commercial amino acid fertilizers across all key traits (fresh weight, plant height, leaf area, soluble protein, chlorophyll; *P* < 0.05), with a particularly marked enhancement in soluble protein (*P* < 0.001). These findings confirmed its potential as a sustainable fertilizer alternative for organic planting systems.

Beyond superior feather-degrading ability, genomic analysis of strain KK^T^ showed 4,022 (59.3%) and 4,106 (60.5%) genes annotated in GO and COG databases, respectively, while KEGG matched only 2,291 genes (33.8%). This indicated abundant uncharacterized genetic resources—functional analysis of which could guide advanced strain development. AntiSMASH 7.0 prediction identified 26 biosynthetic gene clusters (BGCs), 8 more than in *Streptomyces endophytica* sp. nov. (18 BGCs). Among these, 14 BGCs exhibited < 30% similarity to known clusters, with 7 dedicated to antimicrobial peptide synthesis and the remainder putatively assigned to terpenoid, siderophore, hydrogen cyanide (HCN), and melanin biosynthesis. Notably, the clusters for HCN and melanin biosynthesis, which have rarely been reported in *Streptomyces* and are absent in *S. radicis* sp. nov., *S. specialis* GW41-1564^T^, and *S. hoynatensis* strains S1412^T^ and KCTC 29097^T^ [97], may enhance environmental adaptability (e.g., resilience to oxidative stress). In summary, strain KK^T^ exhibited exceptional high-concentration feather degradation efficiency coupled with considerable potential for exploring novel secondary metabolite gene resources.

### The early stress response of KK^T^ strain in feather fermentation medium

Autoclaving (121 °C, 20 min) partially dissolved chicken feathers, releasing soluble protein (1,918.50 µg/mL), free amino acids (135.30 µg/mL), and sulfate (456.00 µg/mL). While these components supported the initial growth of strain KK^T^, they were insufficient to sustain its subsequent proliferation and development.

**Fig. 7:**
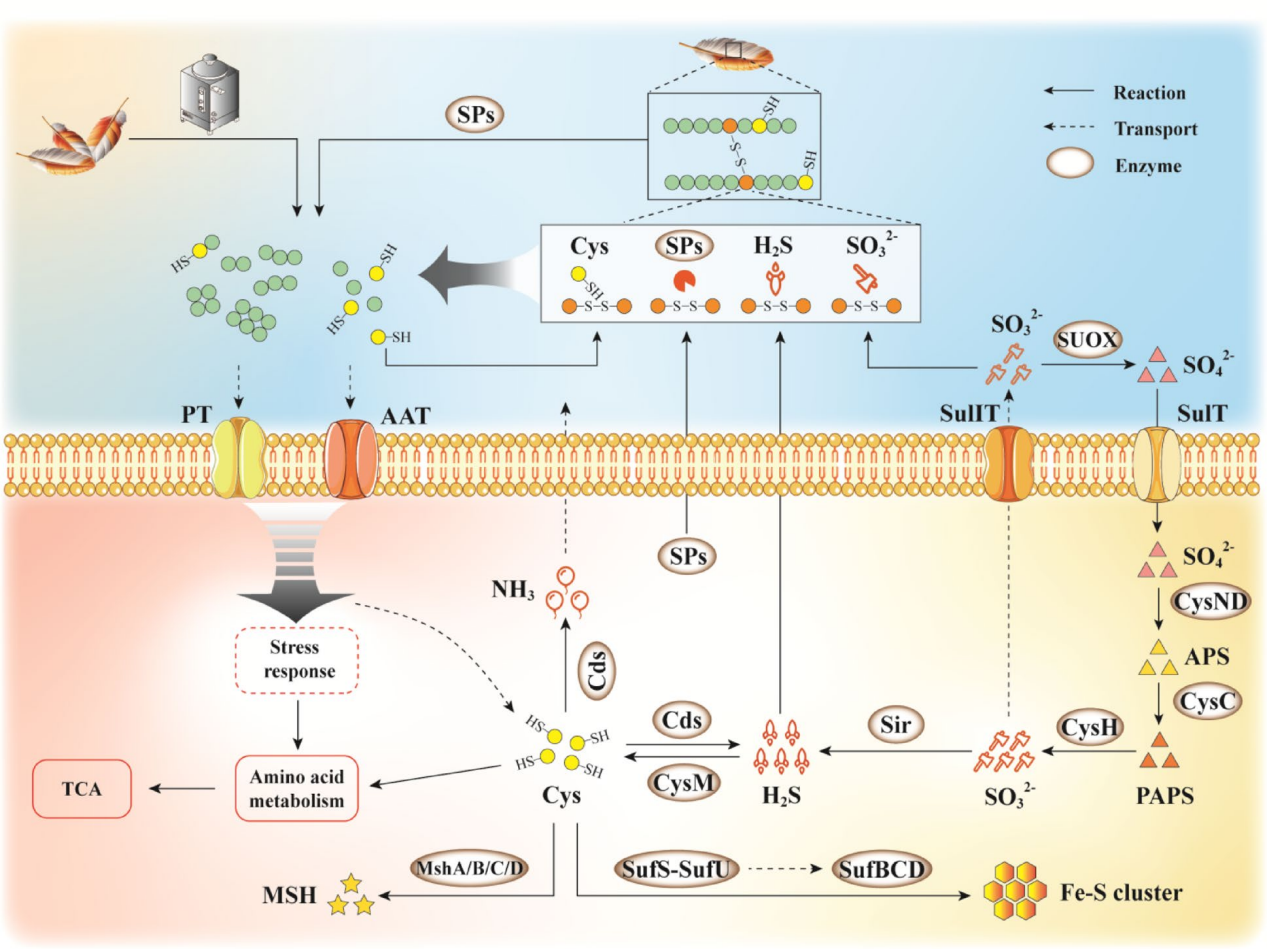
Schematic model of keratin-driven feather degradation by *Streptomyces shaoguanensis* KK^T^. Abbreviations: SPs, secreted proteases; PT, peptides transporter; AAT, amino acid transporter; SulIT, sulfite exporter; SulT, sulfate transport protein

Within the first 0–24 h post-inoculation, strain KK^T^ exhibited a pronounced molecular response, characterized by three key transcriptional signatures: (1) A marked upregulation of 21 genes encoding extracellular proteases was accompanied by significant induction of multiple peptide/amino acid transporters. This indicates that strain KK^T^ rapidly synthesizes and secretes large quantities of extracellular proteases to accelerate the degradation of insoluble keratin into utilizable peptides and amino acids, while enhancing active uptake of these hydrolytic products to support its growth and proliferation. (2) Genes encoding diverse stress-responsive proteins were significantly upregulated, such as Clp protease (a key regulatory enzyme in eubacterial stress responses), redox homeostasis regulators (SOD, AhpC), the small heat shock chaperone Hsp18, the ion-balancing protein YfiZ, osmoprotective transporters (GbuB/BtuD), and translation elongation factors (EF-Tu/EF-Ts). Additionally, genes associated with the Fe-S cluster assembly machinery (the SufBCD scaffold and SufSE sulfur transfer complex) were markedly induced. Notably, Clp protease is well-documented to be upregulated under various stress conditions; it maintains cellular homeostasis and ensures normal metabolic activity by hydrolyzing damaged or misfolded protein aggregates [98]. Of particular relevance, the induction of the Suf system carries important implications. Previous work by Jin et al. on the extreme thermophilic anaerobe *F. islandicum* AW-1 demonstrated that the Suf system not only plays a crucial role in maintaining redox homeostasis and mediating the stress response of Fe-S cluster-containing proteins, but also may be directly involved in keratin degradation [25]. Building on this finding, the coordinated upregulation of the Suf system with genes encoding multiple stress-responsive proteins in strain KK^T^ further suggests that these proteins (including the Suf system) likely constitute the molecular foundation, enabling strain KK^T^ to withstand keratin-induced stress during the early stage of feather degradation. (3) Accompanying the above transcriptional changes, genes involved in core metabolic pathways—including gluconeogenesis, transamination/deamination, the tricarboxylic acid (TCA) cycle, amino acid metabolism, nucleotide metabolism, and oxidative phosphorylation—were also significantly upregulated. This indicates that strain KK^T^ is undergoing extensive metabolic reprogramming to meet the high demands for energy and biosynthetic precursors during cellular growth and proliferation (Fig. 7).

Taken together, these findings demonstrate that, in the nutritionally poor chicken feather medium, strain KK^T^ deploys a suite of coordinated stress response mechanisms to adapt to its environment. These mechanisms include secreting proteases to hydrolyze extracellular proteins, enhancing the transport of peptides and amino acids, clearing intracellular accumulated abnormal proteins via the Clp system, maintaining redox homeostasis through the SOD-AhpC-Suf antioxidant network, and reprogramming central and energy metabolic pathways, collectively ensuring survival and proliferation under nutrient scarcity.

### Mechanism of feather keratin degradation by strain KK^T^

The mechanical pressure hypothesis proposes that filamentous microorganisms (e.g., fungi and actinomycetes) degrade keratin by exerting physical forces or osmotic stress to compromise feather integrity, thereby enhancing protease access to the keratin substrates [99, 100]. Our SEM observations revealed that strain KK^T^ extensively colonized feathers by proliferating hyphae that formed dense entanglements, mechanically disrupting the barbs and barbules. These findings, consistent with reports of filamentous microbes employing physical disruption, indicate that hyphae-generated mechanical forces are a key mechanism of feather degradation by strain KK^T^.

Thiolysis in feather degradation describes microbial-mediated reductive cleavage of keratin disulfide bonds (-S-S-). Microbial metabolism generates small-molecule reducing agents (e.g., sulfite, free cysteine, hydrogen sulfide) and reductive enzymes that collectively mediate nucleophilic cleavage of disulfide bonds. This reaction yields thiol groups (-SH) or sulfonated derivatives (e.g., S-sulfocysteine), thereby destabilizing keratin’s highly cross-linked architecture and facilitating its subsequent proteolytic hydrolysis. Sulfite’s role has garnered particular interest: Li et al. qualitatively demonstrated sustained sulfite accumulation during feather degradation by *Streptomyces* sp. SCUT-3, accompanied by concomitant upregulation of cysteine dioxygenase (CdoI) and sulfite exporter TauE, confirming sulfite-driven disulfide cleavage [18]. Similar co-upregulation of CdoI, Ast1, and TauE in *Bacillus* spp. further supports this mechanism [24, 87]. Strain KK^T^, however, exhibited distinct behavioral patterns: degradation efficiency plateaued by 96 h, yet sulfite remained undetectable during 0–48 h, with only trace levels (2.0–3.2 mg/L) detected at 96–144 h. This pattern of minimal sulfite production mirrors the observation of undetectable free sulfite during wool keratin degradation by *S. fradiae* [26], suggesting that sulfite may not be a critical factor for thiolysis in certain *Streptomyces* strains. Gene expression dynamics revealed two cysteine dioxygenases (critical for sulfite generation) with minimal expression (FPKM < 5) during 0–48 h, followed by significant upregulation at 96 h (FPKM 48.86 and 82.22). Key enzymes in the sulfate reduction pathway (CysD, CysNC, CysC, CysH) were significantly downregulated between 24 and 48 h but upregulated at 96 h; only one sulfate transporter (YbaR) and one sulfite exporter showed upregulation at 96 h. The degradation efficiency of chicken feathers and the free thiol content in the fermentation broth stabilized after 96 h, coinciding with the detection of trace amounts of sulfite. These findings lead us to hypothesize that sulfite may not play a major role in disulfide bond cleavage during the highly efficient degradation of feathers by strain KK^T^.

Hydrolysis of disulfide-rich keratin commonly yields free cysteine, which acts as a key reductant in thiolysis [18, 22]. Studies have shown that intracellular redox homeostasis promotes the release of cysteine and cystine, thereby establishing an extracellular reductive microenvironment [101]. Jin et al. further confirmed that excess intracellular L-cysteine, upon extracellular release, can chemically reduce and cleave keratin disulfide bonds [25]. Genomic analysis of strain KK^T^ revealed no annotated genes encoding cystine/cysteine transporters; however, free cysteine was detected in its feather fermentation broth. This discrepancy suggests two plausible mechanisms for extracellular cysteine accumulation: (i) the presence of uncharacterized transporters that require functional validation; or (ii) more plausibly, the direct generation and retention of free cysteine via extracellular keratin hydrolysis. The resulting extracellular cysteine subsequently functions to reduce disulfide bonds in feather keratin.

Ammonia (NH₃), generated during microbial fermentation, elevates the pH to sustain an alkaline environment that promotes keratin degradation [102]. Under such alkaline conditions, hydrogen sulfide (H₂S) reacts with cystine to yield thiocysteine (Cys-SSH) and cysteine, thereby cleaving disulfide bonds [26]. In this study, strain KK^T^ rapidly produced both NH₃ and H₂S during the early phase of feather degradation. These gases act synergistically: NH₃ establishes and maintains the alkaline milieu necessary for proteolysis, while H₂S directly mediates reductive disruption of disulfide bonds, collectively enabling efficient keratin breakdown.

Enzymatic hydrolysis, the core process in microbial feather keratin degradation, relies on the synergistic action of multiple proteases [1, 19]. Genomic analysis of strain KK^T^ uncovered 208 putative protease genes—surpassing the 149 identified in the feather-degrading *Streptomyces* sp. G11C [103]—of which 61 were significantly upregulated during degradation. Protease expression peaked at the early stage (24 h), with 29 genes highly induced; these were predominantly serine proteases (families S08, S01, S12, S14) and metalloproteases (families M23, M01). Notably, 21 of these upregulated proteases were secreted and are implicated in the initial hydrolysis of keratin. Notably, *KK_01746*, which encodes a papain-like cysteine protease, was significantly upregulated. This represents the first documented instance of such a gene in a feather-degrading microbe and suggests a specific role in the initial degradation phase. Meanwhile, the M06 metalloprotease gene *KK_02604* showed exceptionally high expression at 24 h (FPKM = 4364.49). Given the established role of M06 proteases in keratinolysis [104], its high expression implies a conserved keratin-degrading function for *KK_02604* in strain KK^T^. Later, at the mid-degradation stage (48 h), the expression profile shifted: 6 of the 7 upregulated protease genes encoded intracellular proteases, with only one encoding a secreted enzyme. This pattern coincides with the peak of intracellular peptide processing reported in *B. velezensis* NA16 [82], suggesting a metabolic adaptation focused on the hydrolysis of imported degradation products. At the late degradation stage (96 h), 30 protease genes were significantly upregulated, five of these overlapped with those induced at 24 h (*KK_04748*, *KK_03974*, *KK_05679*, *KK_05039*, *KK_03591*). The reinduction of these secreted proteases likely facilitates the clearance of residual keratin. Collectively, our results reveal that strain KK^T^ orchestrates a triphasic protease expression pattern: (i) an early-stage (24 h) dominated by secreted proteases that initiate extracellular keratinolysis; (ii) a mid-stage (48 h) characterized by the upregulation of intracellular proteases, consistent with the metabolic processing of imported hydrolytic products; and (iii) a late-stage (96 h) featuring the sustained secretion of proteases—including five proteases reinduced from the early stage—to degrade remaining substrates. Thus, this temporal regulation provides the molecular underpinning for efficient and complete feather degradation. However, the specific mechanisms of individual enzymes require further validation through gene manipulation and in vitro biochemical assays.

Based on these findings, we propose that *Streptomyces shaoguanensis* KK^T^ employs a sulfite-independent reductive mechanism, which is potentially mediated by free cysteine, H₂S, and specific reductases, for the cleavage of keratin disulfide bonds. This reductive process is coordinated with a phase-specific protease cascade to achieve efficient keratinolysis. Such an integrated strategy likely represents an adaptive physiological response to limited availability of sulfur, carbon, and nitrogen sources. Within this context, elemental sulfur metabolism—encompassing endogenous sulfur utilization, Fe-S cluster biosynthesis, and redox homeostasis—may serve as a regulatory hub orchestrating metabolic adaptation (the schematic model of the proposed degradation mechanism is depicted in Fig. 7). To decipher the regulatory logic of this process, we are systematically targeting key genes in the proposed mechanism, including those encoding cysteine metabolic enzymes, Fe-S cluster biosynthesis components, disulfide bond reductases, and phase-specific proteases, using gene editing (knockout/overexpression) and heterologous expression. Notably, supplementing with sulfite significantly enhanced the feather degradation percentage to 77.9% (4.0% feather, w/v), compared to 68.6% in sulfur-limited media. This result underscores the critical role of sulfur source modulation in fermentation efficiency. Consequently, subsequent research will focus on the synergistic optimization of sulfur source gradients (e.g., sulfate, cysteine, cystine) and carbon-to-nitrogen (C/N) ratios to pinpoint key regulatory nodes within the sulfur metabolic network and carbon-nitrogen balance, thereby establishing a theoretical basis for developing high-efficiency keratinolytic technologies.

## Conclusion

An actinobacterial strain KK^T^ with remarkable feather-degrading capability was isolated. When cultured with 10.0% (w/v) chicken feathers as the sole nutrient source, it achieved over 50.0% degradation efficiency. This wild-type strain represents one of the few reported isolates capable of maintaining high degradation activity at feather concentrations of ≥ 5.0% (w/v). Based on the 16S rRNA gene similarity, ANI, and dDDH values, along with phylogenetic, phenotypic, and chemotaxonomic characteristics, strain KK^T^ was identified as a novel species within the genus *Streptomyces*, proposed as *Streptomyces shaoguanensis* sp. nov.. The genome revealed substantial biosynthetic potential, harboring 26 biosynthetic gene clusters and a considerable number of genetically uncharacterized putative functional elements. Integrated transcriptomic and metabolomic profiling indicated that feather degradation by strain KK^T^ constitutes an adaptive physiological response to feather-induced stress. The disulfide bond cleavage is mediated through a reductive mechanism primarily dependent on free cysteine, H₂S, and specific reductases—distinct from conventional predominantly sulfite-driven pathways—coupled with temporally coordinated actions of multiple proteases, collectively enabling efficient keratinolysis. Moreover, the feather hydrolysate generated by strain KK^T^ functioned as an effective biofertilizer, significantly promoting the growth of Pak choi. This study provides not only a promising microbial resource for feather biodegradation, but also a solid theoretical foundation for sustainable valorization of feather waste and the development of novel biofertilizers.

### Description of *Streptomyces shaoguanensis* sp. nov

*Streptomyces shaoguanensis* (shao.guan.en’sis, N.L. masc. adj. *shaoguanensis*, pertaining to shaoguan, Guangdong Province, PR China).

Cells are Gram-staining-positive, aerobic actinobacteria that form well-developed, branched substrate and aerial mycelia. Mature aerial hyphae bear long, straight to flexuous chains of cylindrical spores (0.6 × 0.7–1.5 μm) with a textured surface and grey in color. Growth occurs well on ISP1–7, NA, Czapek’s agar, and PDA; melanoid pigments are produced on ISP1, ISP2, ISP6, ISP7, and PDA, while a soluble pink pigment is formed on ISP3. The temperature range for growth is 10–43 ℃ (optimum 30 ℃), and the pH range is 6.0–9.0 (optimum pH 7.0); tolerance to NaCl reaches 5.0% (w/v). The strain utilizes glucose, mannose, xylose, maltose, galactose and fructose as sole carbon sources, and leucine, valine, glutamine, asparagine, threonine, proline, alanine, serine and glycine as sole nitrogen sources. Catalase and oxidase activities are positive. Milk is peptonized and coagulated; H₂S production and hydrolysis of aesculin, urea, starch, and Tween 80 are observed. Nitrate is reduced. The cell wall contains LL-diaminopimelic acid, with galactose and ribose as the characteristic sugars. The polar lipids consist of diphosphatidylglycerol (DPG), phosphatidylethanolamine (PE), phosphatidylinositol (PI), unidentified phosphoglycolipid (PL) and an unidentified aminophospholipid (APL). Menaquinones are MK-10(H₄) and MK-9(H₈). The major fatty acids are anteiso-C₁₅:_0_, iso-C₁₆:_0_, anteiso-C₁₇:_0_, and iso-C₁₅:_0_.

The type strain, KK^T^ (= GDMCC 4.358ᵀ = JCM 36784ᵀ), was isolated from feather waste collected at a poultry market in Shaoguan, Guangdong Province, China. The 16S rRNA gene sequence (accession no. OR539445.1) and the complete genome sequence (7,843,802 bp; G + C content 73.21 mol%; accession no. JASJAK000000000) have been deposited in GenBank.

## Supplementary Information

Supplementary Material 1.

Supplementary Material 2.

## Data Availability

All the data generated during this study are included in this published article.

## Abbreviations

MMT: Million metric tons
TSB: Tryptic Soy Broth
ISP: International Streptomyces Project formulations
NA: Nutrient agar
CZ: Czapek´s sucrose agar
PDA: Potato dextrose agar
SEM: Scanning electron microscopy
TLC: Thin-layer chromatography
HPLC: High-performance liquid chromatography
BGCs: Secondary metabolite biosynthetic gene clusters
dDDH: Digital DNA-DNA hybridization
ANI: Average nucleotide identity
DAP: Diaminopimelic acids
CDSs: Coding sequences
BCA: Bicinchoninic acid
BSA: Bovine serum albumin
DTNB: 5,5´-Dithiobis (2-nitrobenzoic acid)
NTSB: 2-nitro-5-thiosulfobenzoate
CFH: Chicken feather hydrolysate
LL-DAP: LL-diaminopimelic acid
PE: Phosphatidylethanolamine
DPG: Diphosphatidylglycerol
PL: Unidentified phospholipid
PI: Phosphatidylinositol
APL: Unidentified aminophospholipid
COG: Clusters of Orthologous Groups
GO: Gene Ontology
KEGG: Kyoto Encyclopedia of Genes and Genomes
NRPS: Non-ribosomal peptide synthetase
T1PKS: Type I polyketide synthase
RiPP: Post-translationally modified peptide
NAPAA: N-acetyl-para-aminophenol analog
FPKM: Fragments Per Kilobase per Million mapped fragments
CGMCC: China General Microbiological Culture Collection Center
GDMCC: Guangdong Microbial Culture Collection Center
JCM: Japan Collection of Microorganisms
