# Correction: *Streptomyces shaoguanensis* sp. nov.: elucidating the mechanisms of efficient chicken feather degradation and its potential for biofertilizer development

**DOI:** 10.1186/s12934-026-02990-3

**Published:** 2026-03-27

**Authors:** Di Zhou, Weibin Zheng, Yijie Li, Ziqi Zhang, Xia Ding, Ye Ke

**Affiliations:** 1 School of Life Sciences, Nanchang University, Nanchang, 330031 China; 2 School of Biology and Agriculture, Shaoguan University, Shaoguan, 512005 China; 3 Jiangxi Province Key Laboratory of Drug Target Discovery and Validation, Nanchang University, Nanchang, 330031 China

**Correction: Microbial Cell Factories (2026) 25:1** 10.1186/s12934-025-02878-8

In this article [1], under the section **“Description of** ***Streptomyces shaoguanensis*** **sp. Nov”** the JCM deposit number for the type strain has been corrected. For completeness and transparency, the incorrect and correct JCM deposit number for the type strain are displayed below.

**Incorrect JCM deposit number**:

“The type strain, KK^T^ (= GDMCC 4.358^T^ = JCM 36789^T^)”

**Correct JCM deposit number**:

“The type strain, KK^T^ (= GDMCC 4.358^T^ = JCM 36784^T^)”

The original article has been corrected.

## References

[1] Zhou D, Zheng W, Li Y, Zhang Z, Ding X, Ke Y. *Streptomyces shaoguanensis* sp. nov.: elucidating the mechanisms of efficient chicken feather degradation and its potential for biofertilizer development. Microb Cell Fact. 2026;25:1. 10.1186/s12934-025-02878-8 10.1186/s12934-025-02878-8 PMC12763843 41316262

