# Supplementary material for: Streptomyces shaoguanensis sp. nov.: elucidating the mechanisms of efficient chicken feather degradation and its potential for biofertilizer development
Source: Microb Cell Fact. 2025 Nov 28;25:1. doi: 10.1186/s12934-025-02878-8 (PMC12763843; doi:10.1186/s12934-025-02878-8)
Supplement: Supplementary file 2 — Supplementary Material 2. [file 12934_2025_2878_MOESM2_ESM.docx]

**Supplementary Material**

***Streptomyces shaoguanensis* sp. nov.: Elucidating the mechanisms of efficient chicken feather degradation and its potential for biofertilizer development**

**Di Zhou****^1,2^, Weibing Zheng^2^, Yijie Li^2^, Ziqi Zhang^2^, Xia Ding^1^*,** **and Ye Ke^2^***

^1^ School of Life Sciences, Nanchang University, Nanchang 330031, China

^2^ School of Biology and Agriculture, Shaoguan University, Shaoguan 512005, China

^3^Jiangxi Province Key Laboratory of Drug Target Discovery and Validation, Nanchang University, Nanchang 330031, China

***Correspondence:**

Xia Ding

[dingxia97@ncu.edu.cn](mailto:dingxia97@ncu.edu.cn)

Ye Ke

[sgu_keye@sgu.edu.cn](mailto:sgu_keye@sgu.edu.cn), [keye518@126.com](mailto:keye518@126.com)


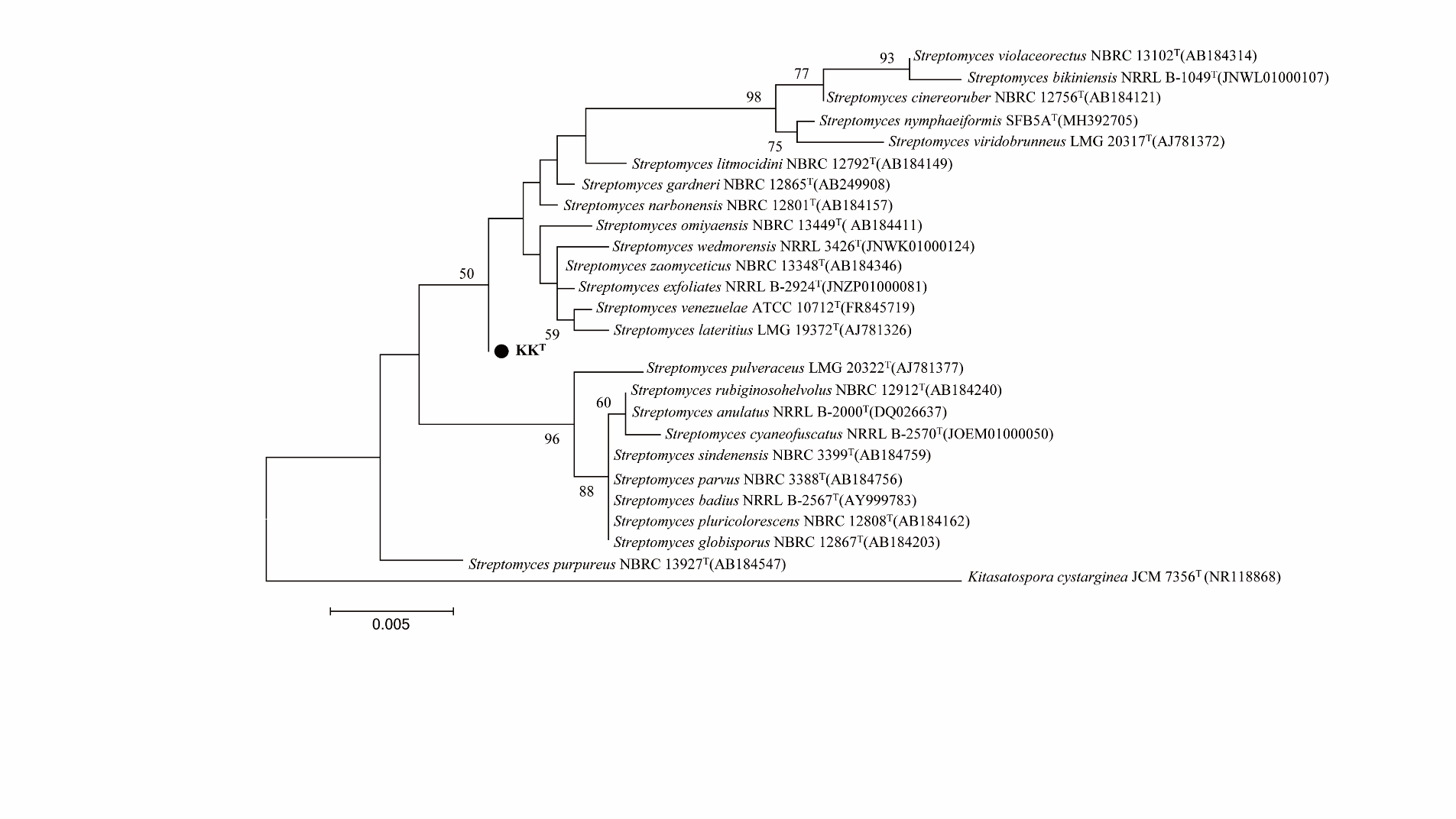


**Supplementary Figure S1. Maximum-Likelihood phylogenetic tree based on 16S rRNA gene sequences from strain KK^T^ and its closely related type strains.** Only bootstrap values above 50% (based on 1000 replications) are indicated. The scale bar indicates 0.005 substitutions per nucleotide position. Strain *Kitasatospora cystarginea* JCM 7356^T^ (NR118868) was used as the out-group.

**
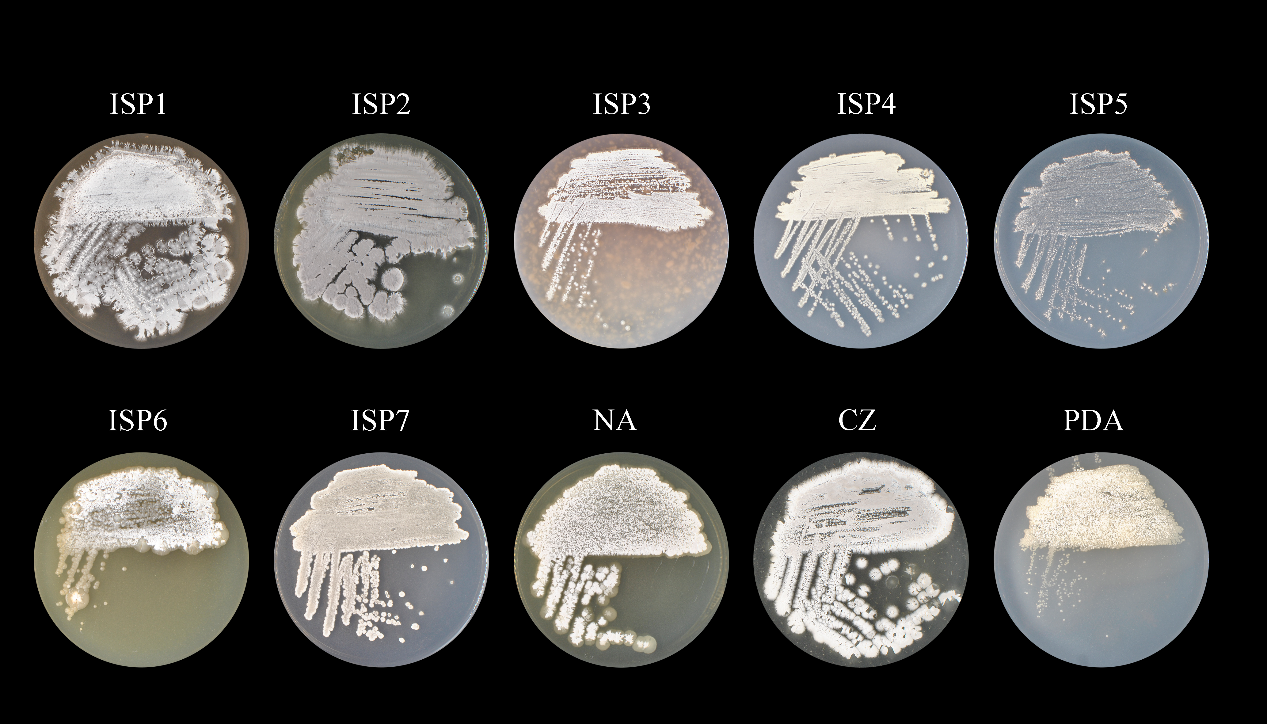
**

**Supplementary Figure S2.** **Morphological features of strain KK^T^ grown on ten different culture media.**

**
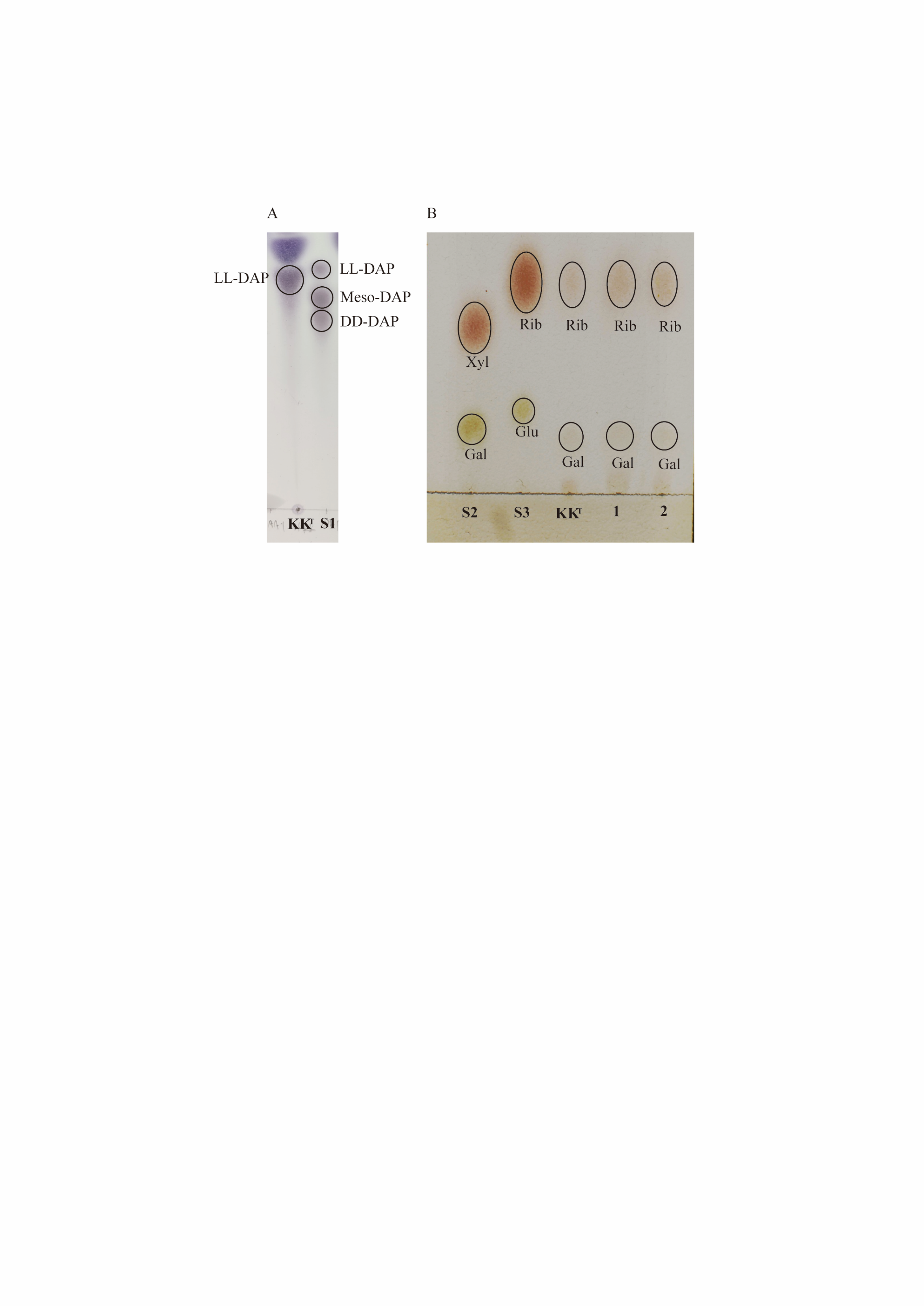
**

**Supplementary Figure S3.** **TLC profiles of whole-cell hydrolysates from strain KK^T^ and its two closest phylogenetic type strains.** S1: DAP (Diaminopimelic acid) reference standard. S2: Xylose (Xyl) and galactose (Gal) mixed reference standards. S3: Ribose (Rib) and glucose (Glu) mixed reference standards. Lane 1: Strain *S.zaomyceticus* CGMCC4.1953^T^. Lane 2: Strain *S.exfoliatus* GDMCC4.124^T^.


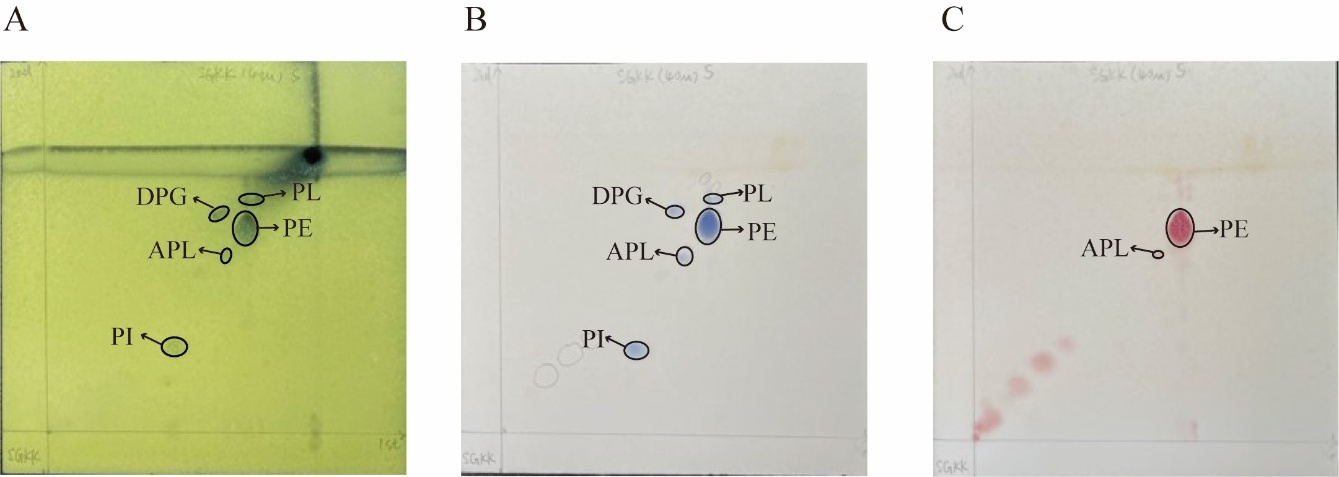


**Supplementary Figure S4.** **2D-TLC analysis of polar lipids from strain KK^T^.** Lipids were visualized using specific staining reagents: (A) phosphomolybdic acid for total lipids; (B) molybdenum blue for phospholipids; (C) ninhydrin for aminolipids. Abbreviations: PE, phosphatidylethanolamine; DPG, diphosphatidylglycerol; PL, unidentified phospholipid; PI, phosphatidylinositol; APL, unidentified aminophospholipid.


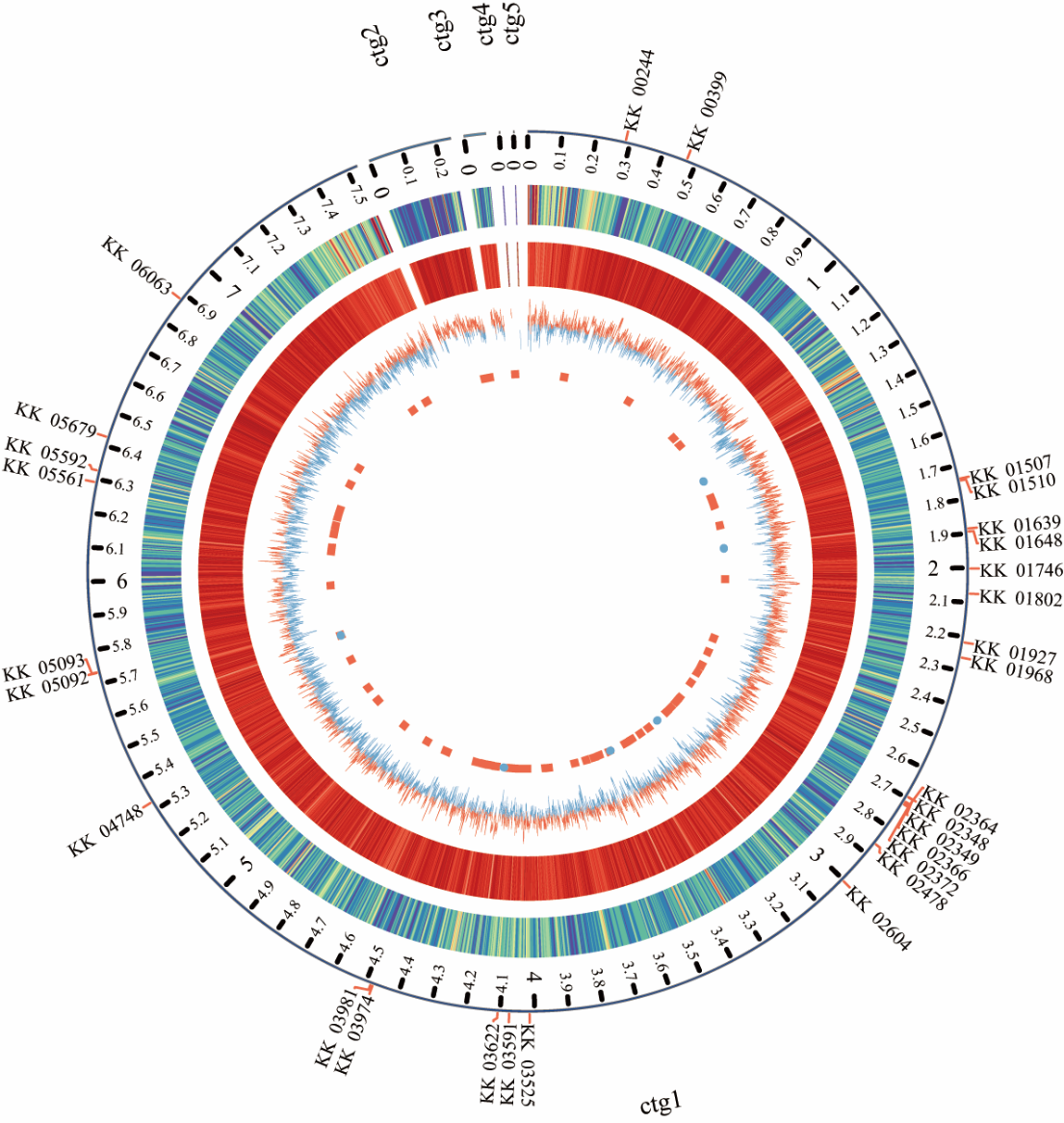


**Supplementary Figure S5.** **Circular genome map of strain KK^T^.** From the outermost to the innermost ring: (1) Genome size (bp), with outer annotations indicating proteases critical for initial feather degradation; (2) Gene density; (3) GC content; (4) GC-skew; and (5) RNA genes (rRNA: blue, tRNA: red).


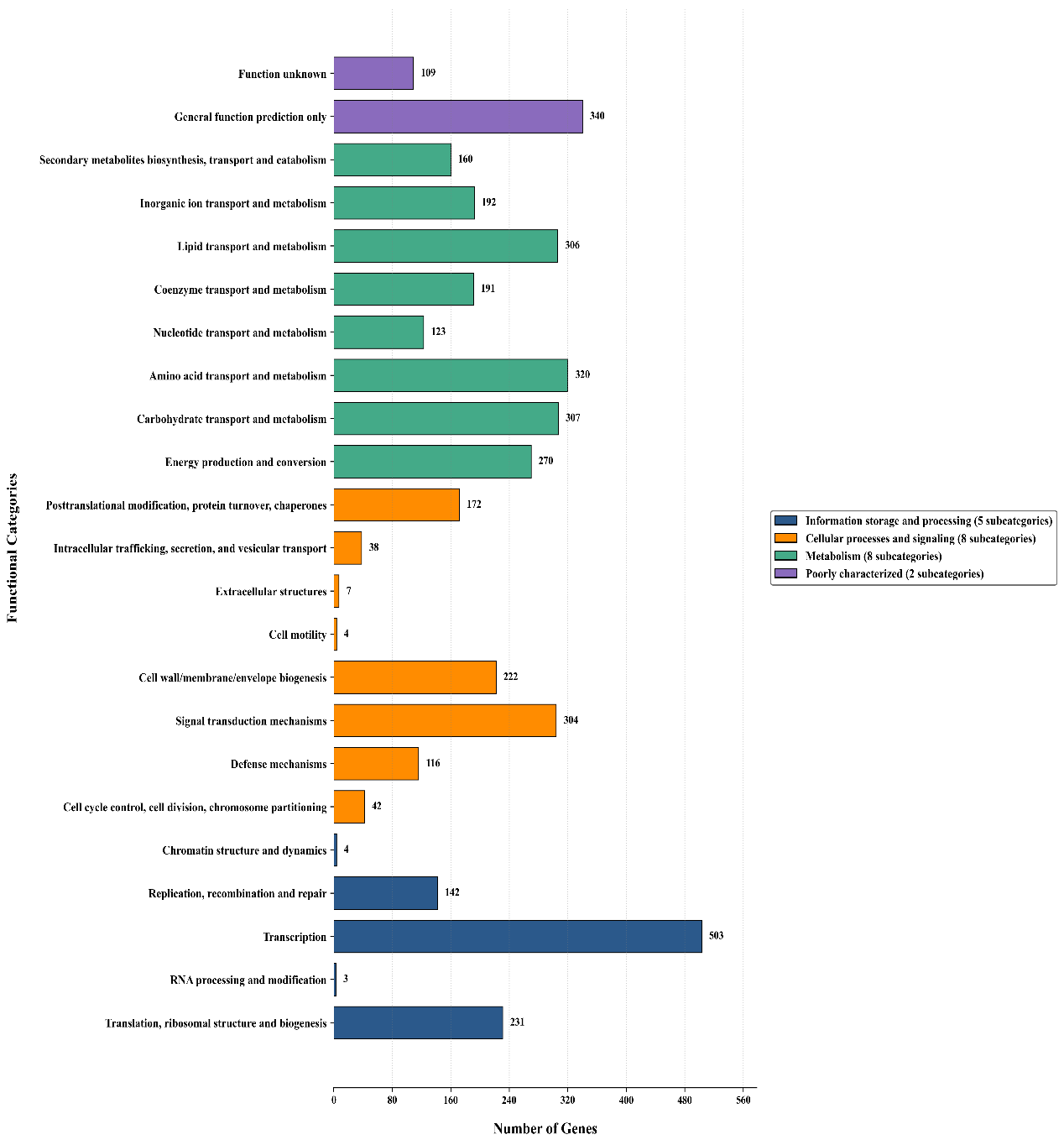


**Supplementary Figure S6.** **COG-based functional classification of annotated genes from strain KK^T^.**


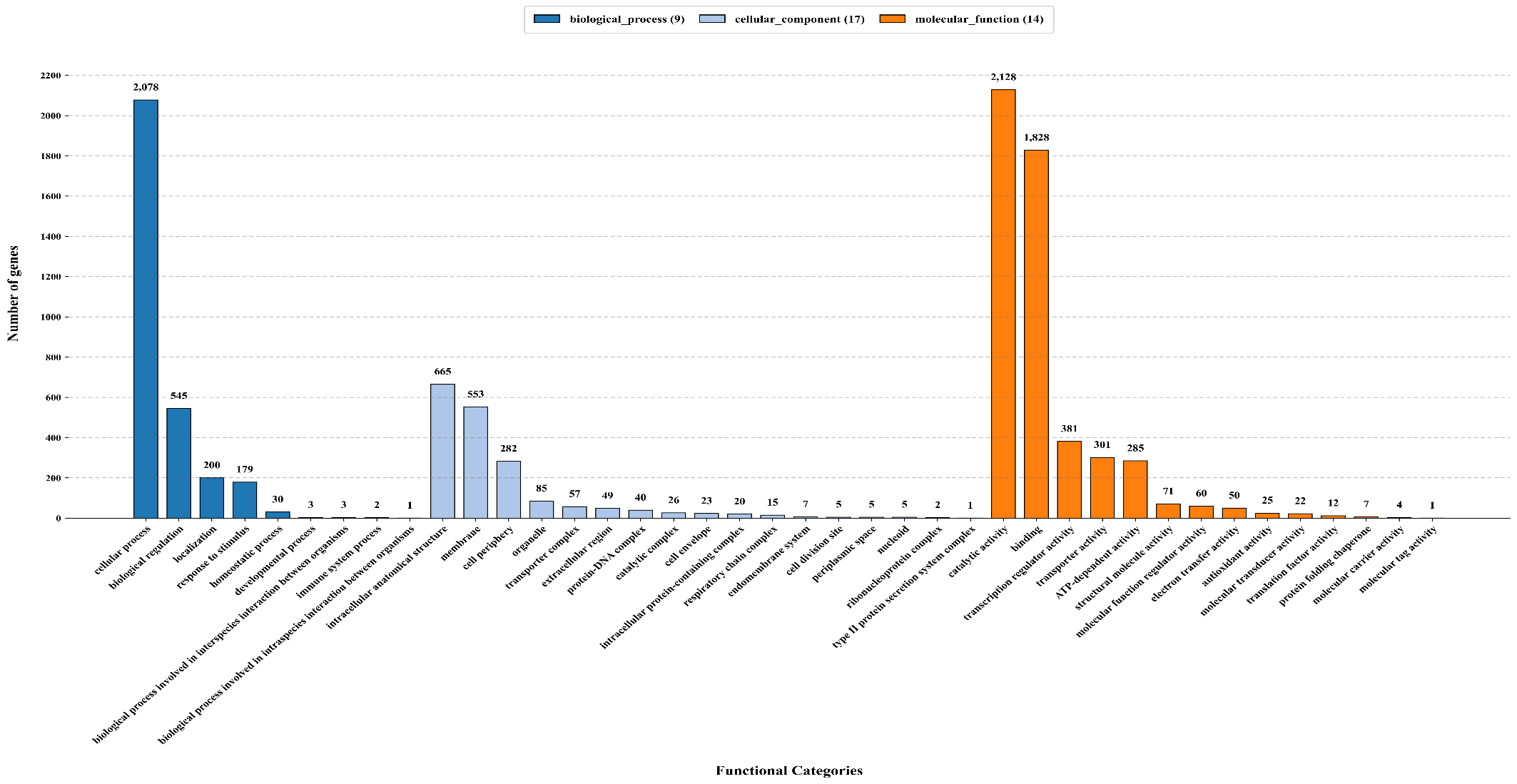


**Supplementary Figure S7. GO-based** **functional categorization of annotated genes from strain KK^T^.**


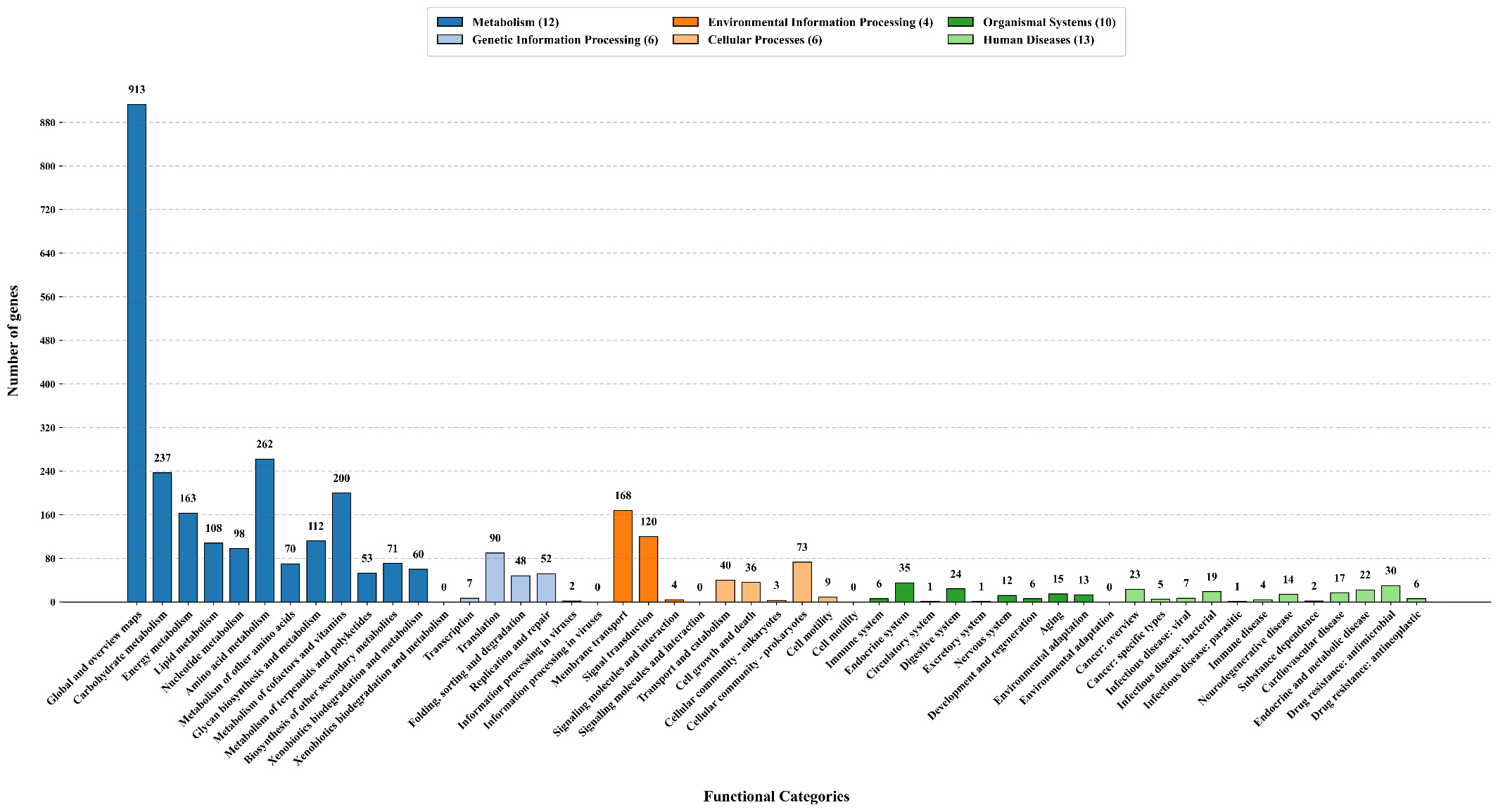


**Supplementary Figure S8. KEGG-based metabolic pathway mapping of annotated genes from strain K****K^T^**
